# Unraveling Rice Tolerance Mechanisms Against *Schizotetranychus oryzae* Mite Infestation

**DOI:** 10.3389/fpls.2018.01341

**Published:** 2018-09-18

**Authors:** Giseli Buffon, Édina Aparecida dos Reis Blasi, Angie Geraldine Sierra Rativa, Thainá Inês Lamb, Rodrigo Gastmann, Janete Mariza Adamski, Joséli Schwambach, Felipe Klein Ricachenevsky, Angelo Schuabb Heringer, Vanildo Silveira, Mara Cristina Barbosa Lopes, Raul Antonio Sperotto

**Affiliations:** ^1^Graduate Program in Biotechnology, Universidade do Vale do Taquari, Lajeado, Brazil; ^2^Biological Sciences and Health Center, Universidade do Vale do Taquari, Lajeado, Brazil; ^3^Graduate Program in Botany, Universidade Federal do Rio Grande do Sul, Porto Alegre, Brazil; ^4^Graduate Program in Biotechnology, Universidade de Caxias do Sul, Caxias do Sul, Brazil; ^5^Graduate Program in Agrobiology, Universidade Federal de Santa Maria, Santa Maria, Brazil; ^6^Graduate Program in Cell and Molecular Biology, Universidade Federal do Rio Grande do Sul, Porto Alegre, Brazil; ^7^Laboratory of Biotechnology, Universidade Estadual do Norte Fluminense “Darcy Ribeiro” (UENF), Campos dos Goytacazes, Brazil; ^8^Integrative Biology Unit, Genomic and Proteomic Facility, Universidade Estadual do Norte Fluminense “Darcy Ribeiro” (UENF), Campos dos Goytacazes, Brazil; ^9^Instituto Rio Grandense do Arroz, Cachoeirinha, Brazil

**Keywords:** phytophagous mite, rice infestation, *Schizotetranychus oryzae*, proteomics, silicon, tolerance

## Abstract

Rice is the staple food for over half of the world’s population. Infestation of *Schizotetranychus oryzae* (Acari: Tetranychidae) causes great losses in rice productivity. To search for rice genotypes that could better tolerate *S. oryzae* infestation, we evaluated morphological and production parameters in Brazilian cultivars, and identified two cultivars with contrasting responses. Leaf damage during infestation was similar for all cultivars. However, infestation in Puitá INTA-CL resulted in reduction in the number of seeds per plant, percentage of full seeds, weight of 1,000 seeds, and seed length, whereas infestation in IRGA 423 increased weight of 1,000 seeds and seed length. Reduction in seed weight per plant caused by infestation was clearly higher in Puitá INTA-CL (62%) compared to IRGA 423 (no reduction detected), thus Puitá INTA-CL was established as susceptible, and IRGA 423 as tolerant to *S. oryzae* infestation. Photosynthetic parameters were less affected by infestation in IRGA 423 than in Puitá INTA-CL, evidencing higher efficiency of energy absorption and use. *S. oryzae* infestation also caused accumulation of H_2_O_2_, decreased cell membrane integrity (indicative of cell death), and accelerated senescence in leaves of Puitá INTA-CL, while leaves of IRGA 423 presented higher levels of total phenolics compounds. We performed proteomics analysis of Puitá INTA-CL and IRGA 423 leaves after 7 days of infestation, and identified 60 differentially abundant proteins (28 more abundant in leaves of Puitá INTA-CL and 32 in IRGA 423). Proteins related to plant defense, such as jasmonate synthesis, and related to other mechanisms of tolerance such as oxidative stress, photosynthesis, and DNA structure maintenance, together with energy production and general metabolic processes, were more abundant in IRGA 423. We also detected higher levels of silicon (as amorphous silica cells) in leaves of infested IRGA 423 plants compared to Puitá INTA-CL, an element previously linked to plant defense, indicating that it could be involved in tolerance mechanisms. Taken together, our data show that IRGA 423 presents tolerance to *S. oryzae* infestation, and that multiple mechanisms might be employed by this cultivar. These findings could be used in biotechnological approaches aiming to increase rice tolerance to mite infestation.

## Introduction

Rice is one of the most important sources for global food security and socioeconomic stability (FAO, 2017). Research directed to this crop are important for the development of technologies that increase productivity and assist farmers who depend on it for subsistence, as is the case in several developing countries such as Brazil (Zeigler and Barclay, 2008), which is the ninth largest rice producer and the main producer outside Asia (FAO, 2017). In the last years, Brazil produced around 10 million tons of rice, with Rio Grande do Sul (RS) state accounting for approximately 70% of this amount. However, monoculture and intensive use of fertilizers benefit the appearance of pest arthropods, which are the main competitors of humans for the resources generated by agriculture (Oerke and Dehne, 2004).

Interactions between plants and herbivores are important determinants of plant productivity in managed and natural vegetation. In response to attack, plants have evolved a range of defenses to reduce the threat of injury and seed set. Crop losses from damage caused by arthropod pests can exceed 15% annually (Mitchell et al., 2016). In order to quantify the pest resistance of the cultivars, the best tool does not seem to be the increase of the arthropod population, but the measurement of the damages caused to the plants, since the reduction of the leaf damage is normally followed by an increase in yield and quality of the grain, and these are the ultimate objectives of most crop breeding programs (Smith, 2005; Erb, 2018). Thus, the plant resistance/tolerance to arthropods is the sum of genetically inherited traits that result in an adapted species that suffers less damage compared to susceptible ones (Stenberg and Muola, 2017). These resistance/tolerance qualities should be measured on a relative scale by comparing levels of damage and productivity with susceptible plants that are severely damaged when exposed to similar experimental conditions (Smith, 2005; Sperotto et al., 2018b). Plant tolerance to arthropods has been indicated as a category of resistance. However, very little is known about the genetic mechanisms of tolerance to arthropods (Peterson et al., 2017).

Tolerance is distinctive in terms of the plant’s ability to withstand or recover from herbivore injury through growth and compensatory physiological processes (Koch et al., 2016; Erb, 2018). Since plant tolerance involves compensatory behavior, the plant is able to bear a large number of herbivores without interfering with the pest’s physiology or behavior (Mitchell et al., 2016; Peterson et al., 2017; Sperotto et al., 2018b). Some studies observed that tolerant plants can compensate photosynthetically by avoiding feedback inhibition and impaired electron flow through PSII that occurs as a result of arthropod feeding. Similarly, the up-regulation of peroxidases and other oxidative enzymes during pest feeding, together with elevated levels of phytohormones, can play an important role in plant tolerance to phytophagous pests (Koch et al., 2016). Tolerance is also currently believed to be caused by other general physiological mechanisms such as pre-existing high levels of carbon storage in roots and increased resource allocation from root to shoot after damage (Peterson et al., 2017).

Phytophagous mites (Acari) comprise a diverse group of arthropods with several species that are pests in crop plants (Blasi et al., 2015). Within this group, the spider mites of the Tetranychidae family are of special interest since they cover a broad host–plant range (Rioja et al., 2017; Blaazer et al., 2018) and can develop into devastating outbreaks (Blasi et al., 2015; Van Leeuwen et al., 2015). Adult spider mites feed from leaves by piercing mesophyll cells with their cheliceral stylets, and sucking the cell content (Villarroel et al., 2016). During feeding, stylets transverse the leaf epidermis either in between epidermal pavement cells or through stomatal openings (Rioja et al., 2017). Such feeding behavior can severely damage leaf tissues (Bensoussan et al., 2016; Rioja et al., 2017). To control such damage there is an indiscriminate use of acaricides. However, mites of the Tetranychidae family have been reported for developing resistance to various acaricides (Osakabe et al., 2016). Physical barriers, such as thick cuticle or wax depositions on the leaf surface (and also around stomatal openings) of some plant hosts impede mites’ ability to penetrate their stylets and feed (Beard et al., 2012; Rioja et al., 2017). Although physical defense plays an important role in preventing mite attack, chemical defenses are recognized as crucial to the plant defense against phytophagous mites (Blasi et al., 2015). Their first chemical defense is to synthesize toxic metabolites (e.g., cyanogenic glycosides, glucosinolates, alkaloids, terpenoids, latex, proteinase inhibitors) with antinutritional, deterrent, repellent, and toxic properties that can reduce plant digestibility for a wide range of potential consumers, and interfere with the metabolism, development, and fecundity of phytophagous mites (Strauss and Zangerl, 2002; Wu and Baldwin, 2010; Mithöfer and Boland, 2012; War et al., 2012; Santamaria et al., 2013, 2018; Rioja et al., 2017; Blaazer et al., 2018). Following this first chemical defense, several proteins are expressed as part of the plant defenses. These molecules are described as anti-insect proteins which negatively affect development or population growth (Blasi et al., 2015). Additionally, several volatiles are produced to attract predators of the phytophagous mites (Blasi et al., 2015; Santamaria et al., 2018). Defense genes involved in the pathways of jasmonic acid (JA), salicylic acid (SA), and ethylene are responsible for the production of defense proteins (glucanases, chitinases, proteases, polyphenol oxidases, protease inhibitors) that can limit the damage of the attacked plant (Blasi et al., 2015; Rioja et al., 2017; Santamaria et al., 2018). Signaling components of the JA and SA pathways can interact with each other, but can also interact with signaling components of growth-regulating hormonal pathways (Pieterse et al., 2012; Blaazer et al., 2018; Sperotto et al., 2018a).

Among the mites from the Tetranychidae family found in rice crops that cause economic damage is *Schizotetranychus oryzae* Rossi de Simons, which has been reported in several South American countries, and generates damages in irrigated rice fields (Ferla et al., 2013). To date, there is little information about the damage and economic loss caused by *S. oryzae* infestation in rice crops, and the available information is usually related to visual effects of the plant. Recently, our group described differentially abundant proteins in rice leaves early infested (EI) (Buffon et al., 2016) and late-infested (LI) (Blasi et al., 2017) by *S. oryzae*, along with the physiological changes induced by such different mite populations. However, the response variability of distinct rice genotypes to *S. oryzae* infestation is unknown, and the molecular and physiological changes caused by infestation in resistant/tolerant and susceptible rice cultivars have not yet been elucidated. Even though *S. oryzae* being the phytophagous mite most commonly found in rice cultivation in the RS state (Ferla et al., 2013), field observations show that some rice cultivars present different levels of infestation, suggesting a possible resistance mechanism. Therefore, we evaluated different rice cultivars commonly cultivated in different regions of RS state aiming to identify different rice responses to *S. oryzae* infestation, in order to understand the molecular and physiological mechanisms behind resistance/tolerance and susceptibility to this mite. Our results may be useful for future breeding programs aiming at resistance/tolerance to phytophagous mite *S. oryzae* infestation.

## Materials and Methods

### Plant Growth Conditions and Mite Infestation

Seeds of rice (*Oryza sativa* L. ssp. *indica*) from IRGA 426, BRS Atalanta, Puitá INTA-CL, IRGA 424, BRS 7 Taim, IRGA 410, and IRGA 423 cultivars were surface sterilized and germinated for 4 days in an incubator (28°C) on paper soaked with distilled water. After germination, plantlets were transferred to vermiculite/soil mixture (1:3) for additional 14 days in greenhouse conditions, and then transferred to plastic buckets containing soil and water. Plastic buckets containing rice plants highly infested by *S. oryzae* were kindly provided by Instituto Rio-Grandense do Arroz (IRGA, Cachoeirinha, RS, Brazil), and were used to infest rice plants in our experiment. Fifty plants (V7-9 stage, according to Counce et al., 2000) of each cultivar (five plants per bucket) were infested by proximity with the bucket containing the highly infested plants placed in the center of the other buckets. For greater homogeneity of infestation and contact, buckets of each cultivar were rotated at a 90° angle counterclockwise every 2 days. Fifty plants of each cultivar were cultivated without infestation (control condition).

The level of damage caused by *S. oryzae* was analyzed from V7-9 stage until the plants reach its final stage of reproductive development (panicle maturity, R9 stage; Counce et al., 2000). Evaluation of damage in the abaxial and adaxial faces of leaves was based on a classification of four levels of infestation: control condition, without any sign of infestation; early infested (EI) leaves, 10–20% of damaged leaf area, average of 168 h of exposure to the mite; intermediate infested (II) leaves, 40–50% of damaged leaf area, average of 360 h; and late infested (LI) leaves, >80% of damaged leaf area, average of 720 h, according to **Supplementary Figure S1**.

### Plant Height and Tiller Number

Plant height and tiller number were evaluated on the seven previously mentioned cultivars during the vegetative stage (V7-9, before being infested) and during the last reproductive stage (R9, control and infested plants). Puitá INTA-CL, BRS 7 Taim, and IRGA 423 cultivars were selected for further analysis based on their different morphological responses to *S. oryzae* infestation.

### Chlorophyll a Fluorescence Transients

The chlorophyll a fluorescence transient was measured on the third upper leaves of control and infested plants of the cultivars Puitá INTA-CL, BRS 7 Taim, and IRGA 423 in three different exposure times: EI, II, and LI, using a portable fluorometer (OS30p, Optisciences, United Kingdom). Before the measurements, plants were dark adapted for 20 min and the fluorescence intensity was measured by applying a saturating pulse of 3,000 μmol photons m^-2^ s^-1^ and the resulting fluorescence of the chlorophyll a measured from 0 to 1 s. The chlorophyll fluorescence intensity rises from a minimum level (the O-level), to a maximum level (the P-level) via two intermediate steps labeled J and I (Stirbet and Govindjee, 2011), also known as OJIP curve (Strasser et al., 2000). These data were used to calculate parameters of the JIP Test (Strasser et al., 2000; Tsimilli-Michael and Strasser, 2008), which are highly studied for *in vivo* investigation of intact photosynthetic apparatus (Jafarinia and Shariati, 2012). Puitá INTA-CL and IRGA 423 cultivars were selected for further analysis based on their different chlorophyll fluorescence responses to *S. oryzae* infestation.

### Seed Analysis

Seeds from Puitá INTA-CL and IRGA 423 cultivars were collected in R9 stage and the following agronomical parameters were evaluated: number of seeds (empty + full) per plant, percentage of full seeds, weight of 1,000 full seeds, and seed length. Yield reduction caused by *S. oryzae* infestation was calculated using the following equation for each cultivar and each condition (control and infested): number of seeds (empty + full) per plant × percentage of full seeds × weight of one seed = seed weight per plant. The seed weight per plant of the infested condition was divided by the seed weight per plant of the control condition, showing an estimative of yield loss percentage in each cultivar caused by *S. oryzae* infestation.

### *In situ* Histochemical Localization of H_2_O_2_ and Loss of Plasma Membrane Integrity

*In situ* accumulation of H_2_O_2_ in control and LI leaves of Puitá INTA-CL and IRGA 423 cultivars was detected by histochemical staining with diaminobenzidine (DAB), according to Shi et al. (2010), with minor modifications. For H_2_O_2_ localization, leaves were immersed in DAB solution (1 mg ml^-1^, pH 3.8) in 10 mM phosphate buffer (pH 7.8), and incubated at room temperature for 8 h in the light until brown spots were visible, which are derived from the reaction of DAB with H_2_O_2_. Leaves were bleached in boiling concentrated ethanol to visualize the brown spots, and kept in 70% ethanol for photo documentation with a digital camera coupled to a stereomicroscope. To determine changes in cell viability (indicative of cell death), another set of control and LI leaves were immersed for 5 h in a 0.25% (w/v) aqueous solution of Evans Blue (Romero-Puertas et al., 2004). Leaves were discolored in boiling concentrated ethanol to develop the blue precipitates, which were photo documented with a digital camera coupled to a stereomicroscope.

### Phenolic Compounds

Phenolic compounds were quantified according to Fett-Neto et al. (1992), with minor modifications. Approximately 50 mg of control and EI leaves from Puitá INTA-CL and IRGA 423 cultivars were pulverized in liquid nitrogen, extracted in 2 ml 0.1 N HCl, and submitted to sonication in a water bath for 30 min. The extracts were centrifuged at 9,000 rpm for 10 min at 4°C. The supernatant was collected and the pellet was re-extracted. The supernatants were pooled and the final volume was completed to 1.5 ml with 0.1 N HCl. For quantification, 300 μl of 20% (w/v) Na_2_CO_3_ and 150 μl of Folin–Ciocalteu reagent were added, mixed, and then incubated at 100°C for 1 min. Absorbance was read at 750 nm. The standard curve was established with gallic acid in 0.1 N HCl.

### Microscope Observation of Amorphous Silica Cells and Silicon Quantification

Control and LI leaves from Puitá INTA-CL and IRGA423 cultivars were used in observation of amorphous silica cells and SiO_2_ quantification. Morphology of silica cells on the leaf surfaces (abaxial and adaxial faces) was observed using SEM. A fresh specimen (0.3–0.5 cm in length) part of the reciprocal fourth leaf was sampled and wiped with tissue paper to remove moisture. The leaf segment was fixed and coated with metal and then loaded onto the SEM. Pictures (at 700× magnification) were obtained to illustrate the differences in amorphous silica cells of rice leaves.

### Plant Protein Extraction and Quantification

Three biological samples (250 mg of fresh matter) of control and EI leaves from Puitá INTA-CL and IRGA 423 cultivars, each containing three leaves from three different plants, were subjected to protein extraction using Plant Total Protein Extraction Kit (Sigma-Aldrich). The protein concentration was measured using 2-D Quant Kit (GE Healthcare, Piscataway, NJ, United States).

### Protein Digestion

For protein digestion, three biological replicates of 100 μg of proteins from Puitá INTA-CL and IRGA 423 leaves were used. Before the trypsin digestion step, protein samples were precipitated using the methanol/chloroform methodology to remove any detergent from samples (Nanjo et al., 2012). Then, samples were resuspended in Urea 7 M and Thiourea 2 M buffer, and desalted on Amicon Ultra-0.5 3 kDa centrifugal filters (Merck Millipore, Germany). Filters were filled to maximum capacity with buffers and centrifuged at 15,000 × *g* for 10 min at 20°C. The washes were performed twice with Urea 8 M and then twice with 50 mM ammonium bicarbonate (Sigma-Aldrich) pH 8.5, remaining approximately 50 μl per sample after the last wash. The methodology used for protein digestion was as previously described (Calderan-Rodrigues et al., 2014). For each sample, 25 μl of 0.2% (v/v) RapiGest^®^ (Waters, Milford, CT, United States) was added, and samples were briefly vortexed and incubated in an Eppendorf Thermomixer^®^ at 80°C for 15 min. Then, 2.5 μl of 100 mM DTT (GE Healthcare, Piscataway, NJ, United States) was added, and the tubes were vortexed and incubated at 60°C for 30 min under agitation. Next, 2.5 μl of 300 mM iodoacetamide (GE Healthcare, Piscataway, NJ, United States) was added, and the samples were vortexed and then incubated in the dark for 30 min at room temperature. The digestion was performed by adding 20 μl of trypsin solution (50 ng/μl; V5111, Promega, Madison, WI, United States) prepared in 50 mM ammonium bicarbonate, and samples were incubated at 37°C during 15 h. For RapiGest^®^ precipitation and trypsin activity inhibition, 10 μl of 5% (v/v) trifluoroacetic acid (TFA, Sigma-Aldrich) was added and incubated at 37°C for 30 min, followed by a centrifugation step of 20 min at 16,000 × *g*. Samples were transferred to Total Recovery Vials (Waters, Milford, CT, United States).

### Mass Spectrometry Analysis

A nanoAcquity UPLC connected to a Synapt G2-Si HDMS mass spectrometer (Waters, Manchester, United Kingdom) was used for ESI–LC–MS/MS analysis. First was performed a chromatography step by injecting 1 μl of digested samples (500 ng/μl) for normalization to relative quantification of proteins. To ensure standardized molar values for all conditions, normalization among samples was based on stoichiometric measurements of total ion counts of MS^E^ scouting runs prior to analyses using the ProteinLynx Global SERVER v. 3.0 program (PLGS; Waters, Milford, CT, United States). Runs consisted of three biological replicates. During separation, samples were loaded onto the nanoAcquity UPLC 5 μm C18 trap column (180 μm × 20 mm) at 5 μl/min during 3 min and then onto the nanoAcquity HSS T3 1.8 μm analytical reversed phase column (75 μm × 150 mm) at 400 nl/min, with a column temperature of 45°C. For peptide elution, a binary gradient was used, with mobile phase A consisting of water (Tedia, Fairfield, OH, United States) and 0.1% formic acid (Sigma-Aldrich), and mobile phase B consisting of acetonitrile (Sigma-Aldrich) and 0.1% formic acid. Gradient elution started at 7% B, then ramped from 7% B to 40% B up to 91.12 min, and from 40% B to 99.9% B until 92.72 min, being maintained at 99.9% until 106.00 min, then decreasing to 7% B until 106.1 min and kept 7% B until the end of experiment at 120.00 min. Mass spectrometry was performed in positive and resolution mode (V mode), 35,000 FWHM, with ion mobility, and in data-independent acquisition (DIA) mode; ion mobility separation (HDMS^E^) using IMS wave velocity of 600 m/s, and helium and IMS gas flow of 180 and 90 ml/min, respectively; the transfer collision energy ramped from 19 to 55 V in high-energy mode; cone and capillary voltages of 30 and 2750 V, respectively; and a source temperature of 70°C. In TOF parameters, the scan time was set to 0.5 s in continuum mode with a mass range of 50–2,000 Da. The human [Glu1]-fibrinopeptide B (Sigma-Aldrich) at 100 fmol/μl was used as an external calibrant and lock mass acquisition was performed every 30 s. Mass spectra acquisition was performed by MassLynx v4.0 software.

### Bioinformatics Analysis

Spectra processing and database searching conditions were performed by Progenesis QI for Proteomics Software V.2.0 (Nonlinear Dynamics, Newcastle, United Kingdom). The analysis used the following parameters: Apex3D of 150 counts for low energy threshold, 50 counts for elevated energy threshold, and 750 counts for intensity threshold; one missed cleavage, minimum fragment ion per peptide equal to two, minimum fragment ion per protein equal to five, minimum peptide per protein equal to two, fixed modifications of carbamidomethyl (C) and variable modifications of oxidation (M) and phosphoryl (STY), and a default false discovery rate (FDR) value at a 1% maximum, peptide score greater than four, and maximum mass errors of 10 ppm. The analysis used the *O. sativa* protein databank from Phytozome^1^. Label-free relative quantitative analyses were performed based on the ratio of protein ion counts among contrasting samples. After data processing and to ensure the quality of results, only proteins present or absent (for unique proteins) in three out of three runs were accepted and submitted to differentially abundance analysis. Proteins were considered to be up-regulated if the fold change (FC) was greater than 1.5 and down-regulated if the FC was less than 0.6667, and both with significantly *P*-value ANOVA (*P* < 0.05). The Blast2GO tool^2^ was used to identify proteins with known Gene Ontology (GO) annotations (Conesa et al., 2005). Detected proteins were also analyzed using the B2G Kegg maps (Ashburner et al., 2000).

### RNA Extraction and cDNA Synthesis

Total RNA was extracted from control and EI rice leaves of Puitá INTA-CL and IRGA 423 cultivars using NucleoSpin RNA Plant (Macherey-Nagel). First-strand cDNA synthesis was performed using the SMART PCR cDNA Synthesis Kit (Clontech Laboratories, Mountain View, CA, United States) with reverse transcriptase (M-MLV, Invitrogen, Carlsbad, CA, United States) and 2 μg of RNA quantified using Qubit RNA Assay Kit (Invitrogen, Carlsbad, CA, United States) and Qubit 2.0 Fluorometer.

### Quantitative RT-PCR and Data Analysis

RT-qPCRs were carried out in a StepOne Real-Time Cycler (Applied Biosystems). All primers (listed in **Supplementary Table S1**) were designed to amplify 100–150 bp of the 3′-UTR of the genes (*2,3-bisphosphoglycerate-independent phosphoglycerate mutase*, *hexokinase*, and *glutathione reductase*) and to have similar Tm values (60 ± 2°C). Reaction settings were composed of an initial denaturation step of 5 min at 94°C, followed by 40 cycles of 10 s at 94°C, 15 s at 60°C, 15 s at 72°C, and 35 s at 60°C (fluorescence data collection); samples were held for 2 min at 40°C for annealing of the amplified products and then heated from 55 to 99°C with a ramp of 0.1°C/s to produce the denaturing curve of the amplified products. RT-qPCRs were carried out in 20 μl final volume composed of 10 μl of each reverse transcription sample diluted 100 times, 2 μl of 10× PCR buffer, 1.2 μl of 50 mM MgCl_2_, 0.1 μl of 5 mM dNTPs, 0.4 μl of 10 μM primer pairs, 4.25 μl of water, 2.0 μl of SYBRGreen (1:10,000, Molecular Probe), and 0.05 μl of Platinum Taq DNA Polymerase (5 U/μl, Invitrogen, Carlsbad, CA, United States). Gene expression was evaluated using a modified 2^-ΔCT^ method (Schmittgen and Livak, 2008), which takes into account the PCR efficiencies of each primer pair (Relative expression TESTED GENE/CONTROL GENE = (PCR_eff_ CG)^CtCG^/(PCR_eff_ TG)^CtTG^). *OsUBQ5* gene expression was used as an internal control to normalize the relative expression of tested genes (Jain et al., 2006). Each data point corresponds to three biological and four technical replicate samples. The expression of a senescence marker gene (*Staygreen* gene, *OsSGR*, a chloroplast protein which regulates chlorophyll degradation by inducing LHCII disassembly through direct interaction; Park et al., 2007) was also analyzed in control and early/intermediate infested leaves.

### Statistical Analysis

Data were analyzed using the Student’s *t*-test (*P* ≤ 0.05, 0.01, and 0.001) or One-Way ANOVA followed by Tukey’s test, using SPSS Base 21.0 for Windows (SPSS Inc., United States).

## Results

### Different Physiological Responses of Rice Cultivars to *S. oryzae* Infestation

**FIGURE 1:**
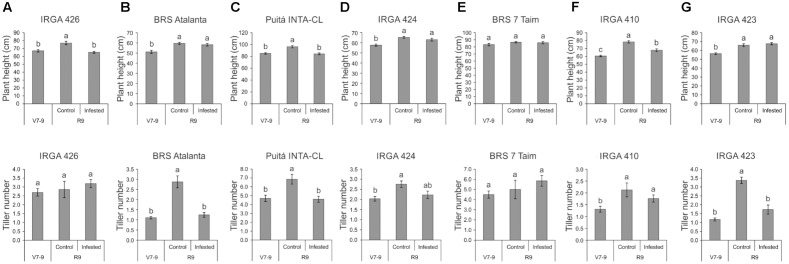
Plant height (cm) and tiller number of the seven tested cultivars, **(A)** IRGA 426, **(B)** BRS Atalanta, **(C)** Puitá INTA-CL, **(D)** IRGA 424, **(E)** BRS 7 Taim, **(F)** IRGA 410, and **(G)** IRGA 423, at the vegetative stage (no infestation, V7-9) and full maturity stage (control or infested conditions, R9). Represented values are the averages of 50 samples ± SE. Different letters indicate that the means are different by the Tukey’s HSD test (*P* ≤ 0.05).

The first screening of rice responses to *S. oryzae* infestation showed that all tested cultivars present similar pattern of infestation kinetics (**Supplementary Figure S2**). After 5 weeks, high infestation levels were detected in all cultivars. Therefore, none of the tested cultivars seems to be resistant to *S. oryzae* infestation. On the other hand, plant height and tiller number were differentially affected by mite infestation (**Figure 1**). *S. oryzae* affected the plant growth of IRGA 426, Puitá INTA-CL, and IRGA 410 cultivars (**Figures 1A,C,F**), and also the tillering in BRS Atalanta, Puitá INTA-CL, and IRGA 423 cultivars (**Figures 1B,C,G**). Even though we were not able to find any sign of resistance in these cultivars, we decided to further characterize the response to *S. oryzae* of three cultivars that showed different responses to mite infestation. Therefore, we selected Puitá INTA-CL, BRS7 Taim, and IRGA 423 cultivars to further analysis. Even though BRS Atalanta cultivar presented similar response of IRGA 423 (plant height not affected and tiller number deeply affected by infestation), we selected IRGA 423 for being commonly considered as more productive in southern Brazilian properties.

**FIGURE 2:**
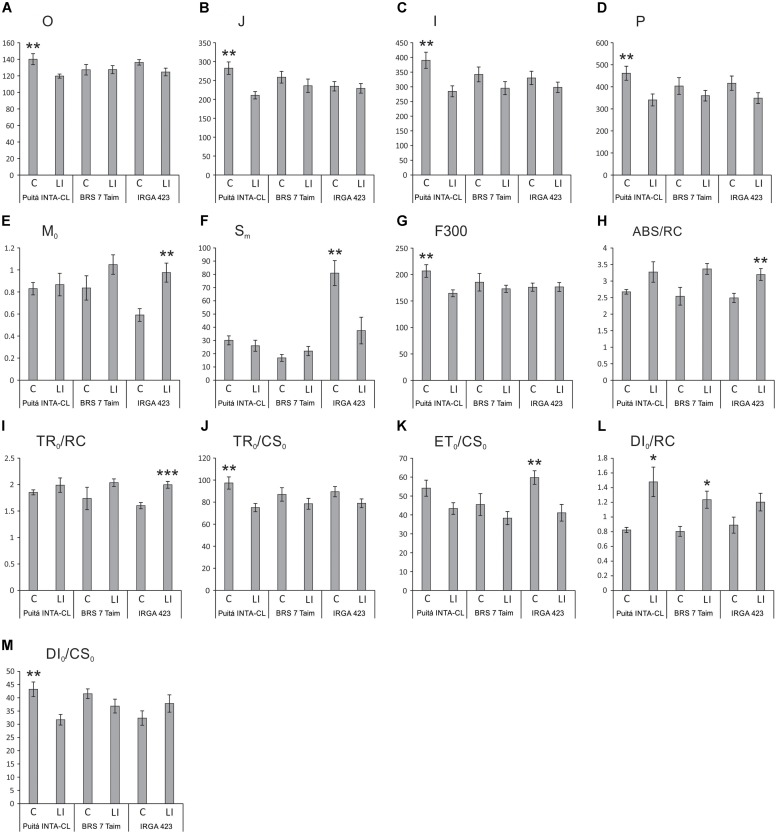
OJIP-test parameters calculated from the chlorophyll a fluorescence transient in control (C) and late infested (LI) leaves of the three tested cultivars, (Puitá INTA-CL, BRS 7 Taim, and IRGA 423). **(A)** O, **(B)** J, **(C)** I, **(D)** P, **(E)** M_0_, **(F)** S_m_, **(G)** F300, **(H)** ABS/RC, **(I)** TR_0_/RC, **(J)** TR_0_/CS_0_, **(K)** ET_0_/CS_0_, **(L)** DI_0_/RC, and **(M)** DI_0_/CS_0_. Represented values are the averages of 10 samples ± SE. Mean values (from each cultivar) with one, two, or three asterisks are significantly different as determined by the Student’s *t*-test (*P* ≤ 0.05, 0.01, and 0.001, respectively).

Chlorophyll a fluorescence analysis showed that EI and II conditions were not enough to change any parameter on the three tested cultivars (data not shown). On the other hand, several parameters were affected by LI condition. Puitá INTA-CL decreased the energy flow in PSII throughout the four OJIP curve-times when comparing control and LI plants, while both Puitá INTA-CL and BRS 7 Taim did not show any decrease in the same parameter (**Figures 2A–D**). On the other hand, IRGA 423 LI plants increased the net rate of reaction centers closure (M_0_), reducing the energy needed to close all the reaction centers of the thylakoidal membrane (S_m_), thus showing greater efficiency in energy use, while Puitá INTA-CL and BRS 7 Taim did not present any difference compared to control plants (**Figures 2E,F**). Also, Puitá INTA-CL LI plants reduced the fluorescence intensity at F300 (0.30 ms intensity, at the maximum point of fluorescence emission), where the plastoquinone is reduced and the reaction centers are closed, showing that this cultivar will have less energy to be used in the next phases of photosynthesis, whereas the other two cultivars present no changes in the same parameter (**Figure 2G**).

The energy absorption per reaction center (ABS/RC) is significantly increased only in infested leaves of IRGA 423 cultivar (**Figure 2H**), evidencing that these plants try to obtain more energy to tolerate the herbivory stress. The light energy capture per reaction center (TR_0_/RC), which is converted in chemical energy during photosynthesis, is also increased only in infested leaves of IRGA 423 cultivar (**Figure 2I**). Puitá INTA-CL was the only cultivar that decreased light energy capture per active leaf area (TR_0_/CS_0_ – **Figure 2J**). The electron transport per active leaf area (ET_0_/CS_0_) decreased in infested leaves of IRGA 423 cultivar (**Figure 2K**), probably decreasing ATP production as a mean of favoring defense over energy production in this cultivar. Puitá INTA-CL and BRS 7 Taim presented increased energy dissipation per reaction center (DI_0_/RC – **Figure 2L**), evidencing that these two cultivars are more affected by *S. oryzae* infestation than IRGA 423, due to less efficient energy use. Puitá INTA-CL also presented a decrease in energy dissipation per active leaf area (DI_0_/CS_0_ – **Figure 2M**). Based on these data, we concluded that Puitá INTA-CL and IRGA 423 show contrasting responses to *S. oryzae* infestation. Therefore, we selected these cultivars for further analysis.

**FIGURE 3:**
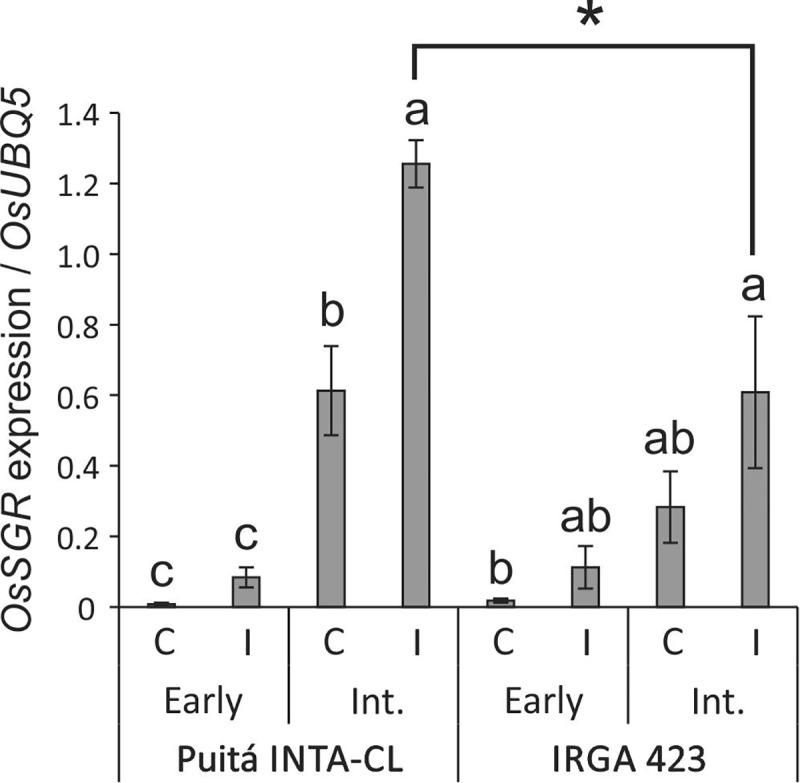
*OsSGR* expression in control and early and intermediate infested leaves of the two tested cultivars (Puitá INTA-CL and IRGA 423). Represented values are the averages of three samples ± SE. Different letters indicate that the means (from each cultivar) are different by the Tukey’s HSD test (*P* ≤ 0.05). Mean values with one asterisk are significantly different as determined by the Student’s *t*-test (*P* ≤ 0.05).

The impaired chlorophyll fluorescence of Puitá INTA-CL suggests that *S. oryzae* infestation can promote an earlier senescence process on the leaves of this cultivar when compared to IRGA 423. Such hypothesis was confirmed by the higher expression of *OsSGR* gene (a senescence marker) in II leaves of Puitá INTA-CL (**Figure 3**).

**FIGURE 4:**
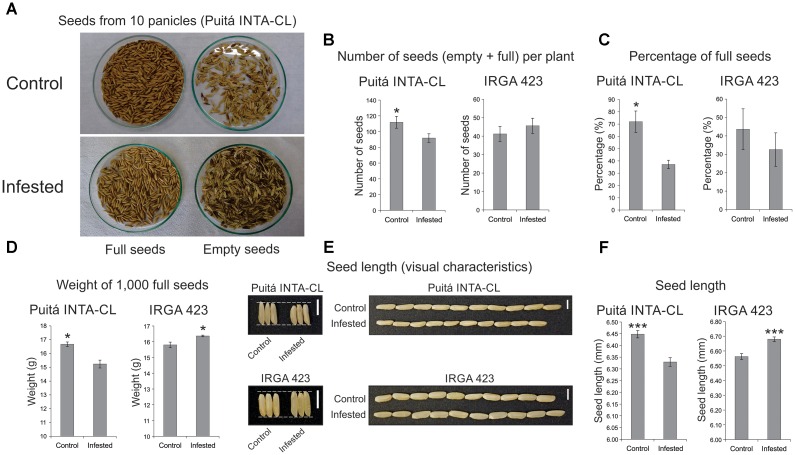
Seeds analysis of the two tested cultivars (Puitá INTA-CL and IRGA 423). **(A,B)** Number of seeds (empty + full) per plant. **(C)** Percentage of full seeds. **(D)** Weight of 1,000 full seeds. **(E)** Seed length (visual characteristics). **(F)** Seed length (mm). Represented values are the averages of 50 samples ± SE. Mean values with one or three asterisks are significantly different as determined by the Student’s *t*-test (*P* ≤ 0.05 and 0.001). Bars in panel **(E)** indicate 0.5 cm.

**FIGURE 5:**
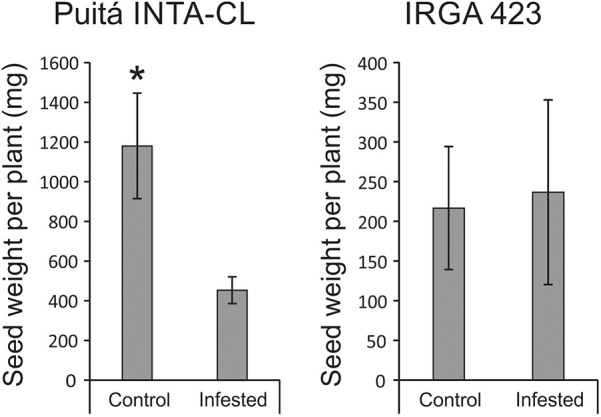
Seed weight per plant (estimative of yield) of the two tested cultivars (Puitá INTA-CL and IRGA 423). Represented values are the averages of 10 samples ± SE. Mean values with one asterisk are significantly different as determined by the Student’s *t*-test (*P* ≤ 0.05).

Seeds from both cultivars were evaluated in order to verify whether *S. oryzae* infestation can decrease rice yield. As seen in **Figure 4**, seeds from Puitá INTA-CL cultivar were more affected by *S. oryzae* infestation than seeds from IRGA 423, showing a decrease in the number of seeds per plant (**Figures 4A,B**), percentage of full seeds (**Figure 4C**), weight of 1,000 full seeds (**Figure 4D**), and seed length (**Figures 4E,F**), resulting in approximately 62% reduction in seed weight per plant, which is an estimate of yield loss (**Figure 5**). On the other hand, seeds from IRGA 423 presented an increase in the weight of 1,000 full seeds (**Figure 4D**), explained by an increase in seed length (**Figures 4E,F**), resulting in no yield loss (**Figure 5**). Based on these data, we suggest that Puitá INTA-CL is susceptible to *S. oryzae* infestation, while IRGA 423 can be considered tolerant. From now on, we will call Puitá INTA-CL and IRGA 423 as “susceptible” and “tolerant” cultivars, respectively.

**FIGURE 6:**
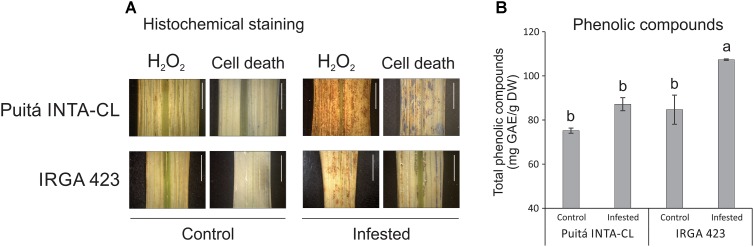
Histochemical staining assay **(A)** of H_2_O_2_ and loss of plasma membrane integrity (indicative of cell death), by DAB and Evans Blue, respectively, in control and LI leaves of the two tested cultivars (Puitá INTA-CL and IRGA 423). The positive staining (detected in higher levels on infested leaves) in the photomicrographs shows as bright images (brown-color for DAB and blue-color for Evans Blue). Bars in figures indicate 0.5 cm. Total phenolic compounds **(B)** of control and EI leaves of the two tested cultivars (Puitá INTA-CL and IRGA 423). Represented values are the averages of three samples ± SE. Different letters indicate that the means are different by the Tukey’s HSD test (*P* ≤ 0.05). GAE, gallic acid equivalents; DW, dry weight.

As seen in **Figure 6A**, leaves of the tolerant cultivar accumulate lower levels of H_2_O_2_ and less evidence of cell death (higher level of plasma membrane integrity) than the susceptible cultivar. Therefore, *S. oryzae* infestation differentially affect the generation of oxidative stress and the cell death level on the LI leaves of susceptible and tolerant cultivars. Such low levels of oxidative stress on the leaves of tolerant cultivar could be explained, at least partially, by the higher level of phenolic compounds on the infested leaves of tolerant cultivar when compared to susceptible one (**Figure 6B**).

**FIGURE 7:**
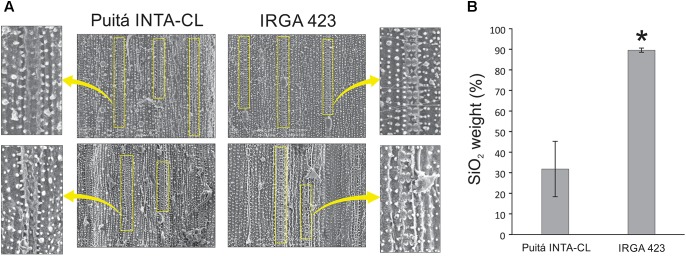
Microscope observation of amorphous silica cells and SiO_2_ quantification. **(A)** SEM of the infested leaf surfaces (adaxial face) from susceptible Puitá INTA-CL and tolerant IRGA 423 cultivars, highlighting the amorphous silica cells. **(B)** Quantification of SiO_2_ using SEM. Figures in **(A)** are representatives of 10 analyzed leaf surfaces from each cultivar. Represented values in **(B)** are the averages of 10 samples ± SE. Mean values with one asterisk are significantly different as determined by the Student’s *t*-test (*P* ≤ 0.05). DW, dry weight.

We used SEM to visualize the leaf surfaces of susceptible and tolerant plants during *S. oryzae* infestation. Under control condition, both cultivars presented similar levels of amorphous silica cells on the abaxial face and diminute amounts on the adaxial face (data not shown). However, under infested condition, adaxial face of tolerant IRGA 423 cultivar presents higher levels of amorphous silica cells than the susceptible Puitá INTA-CL (**Figure 7A**), while similar amounts were found on the abaxial face (data not shown). Also, adaxial surface of the tolerant cultivar accumulates more SiO_2_ (major component of the amorphous silica cells) than the susceptible one (**Figure 7B**).

### Overview of Proteomic Analysis

A crucial step in plant defense is the early perception of stress in order to respond quickly and efficiently (Rejeb et al., 2014). A total of 728 proteins were identified comparing control and infested conditions in both cultivars, with 332 (45.6%) unique to or differentially abundant between cultivars. As seen in **Supplementary Figure S3**, comparing control and infestation leaves of susceptible cultivar, we detected 118 proteins, being 63 more abundant (and one unique) in control condition and 54 more abundant in infested condition. We identified 217 proteins in control and infestion conditions of tolerant cultivar, being 84 more abundant (and two uniques) in control condition and 82 more abundant (and one unique) in infested condition. When we compared both cultivars in control condition, we identified 137 proteins, with 97 more abundant (and one unique) in susceptible cultivar and 96 more abundant in tolerant one. Comparison of both cultivars in infested condition generated 60 proteins, with 28 more abundant in susceptible cultivar and 32 more abundant in tolerant one.

The corresponding sequence of each identified protein was compared to NCBI using BLASTp to identify specific domains, molecular functions, and protein annotations. Afterward, proteins were categorized in functional categories, according to its putative molecular function. The lists of all unique or differentially abundant proteins identified in this work are presented in **Supplementary Tables S2**–**S5**.

Several metabolic processes seem to be inhibited by *S. oryzae* infestation on the susceptible Puitá INTA-CL cultivar, including translation, carbohydrate metabolism and energy production (especifically glycolysis), photosynthesis, and response to stress. On the other hand, the higher abundance of oxidative stress- and ATP synthesis-related proteins in infected leaves suggest an attempt to respond to *S. oryzae* infestation (**Supplementary Table S2**). On the tolerant IRGA 423, *S. oryzae* infestation seems to be less damaging and to generate a more complex defense response. Several proteins involved with protein modification and degradation, general metabolic processes, carbohydrate metabolism and energy production (especially galactose and polysaccharide metabolism), oxidative stress, response to stress, photosynthesis, amino acid metabolism, and DNA structure maintenance were identified as more abundant in infested than in control condition. Even though, some categories are still inhibited by infestation, as translation, transport, and lipid metabolism (**Supplementary Table S3**). Surprisingly, when we compare both cultivars in control condition, the susceptible Puitá INTA-CL seems to present all the metabolic processes more active than the tolerant IRGA 423 (**Supplementary Table S4**). However, when both cultivars are compared in infested conditions, the tolerant IRGA 423 presents higher expression of proteins related to carbohydrate metabolism/energy production and general metabolic processes than the susceptible Puitá INTA-CL, which in turn, shows increased expression of proteins related to translation and transport. Also, under infested condition, the susceptible cultivar seems to prioritize growth over defense, due to the higher expression of a gibberelin (GA) receptor, while the tolerant one seems to prioritize defense over growth, due to the higher expression of jasmonate *O*-methyltransferase (**Supplementary Table S5**), a key enzyme for jasmonate-regulated plant responses.

### GO Enrichment and KEGG Pathways

Gene Ontology analysis provided an overview of rice molecular response to *S. oryzae* infestation in susceptible and tolerant plants. The GO annotations of all 332 differentially abundant and unique proteins identified are shown in **Supplementary Figures S4**, **S5**. As expected, several biological processes are regulated when control and infested conditions are compared in both cultivars (control condition: Puitá INTA-CL × IRGA 423; infested condition: Puitá INTA-CL × IRGA 423), with a higher number of regulated biological processes during infestation. Two biological processes (cellular component organization and regulation of cellular processes) are more regulated on the tolerant cultivar (IRGA 423: control × infested) when compared to the susceptible one, and could be related to a more efficient plant defense (**Supplementary Figure S4**). The molecular function of structural constituent of ribosome is only regulated on the susceptible cultivar (Puitá INTA-CL: control × infested), and the protein binding is only regulated on the tolerant one (IRGA 423: control × infested) (**Supplementary Figure S5**).

To identify specific pathways affected by *S. oryzae* infestation in susceptible and tolerant rice plants, we also analyzed KEGG pathways. The following KEGG pathways (involving five or more proteins) were identified as associated with proteins differentially abundant only on the tolerant IRGA 423 cultivar (control × infested conditions): pyruvate metabolism (11), glyoxylate and dicarboxylate metabolism (8), amino sugar and nucleotide sugar metabolism (8), and methane metabolism (5). On the other hand, the following KEGG pathways (involving five or more proteins) were identified as associated with proteins differentially abundant in control condition (susceptible Puitá INTA-CL × tolerant IRGA 423): Glyoxylate and dicarboxylate metabolism (8), citrate cycle (TCA cycle) (5), starch and sucrose metabolism (5), carbon fixation in photosynthetic organisms (5), amino sugar and nucleotide sugar metabolism (5), and frutose and mannose metabolism (5). The only pathway identified as associated with proteins differentially abundant in infested condition (susceptible Puitá INTA-CL × tolerant IRGA 423) is gycolysis/gluconegenesis (5), suggesting a complete different pattern of energy use employed by the cultivars in both tested conditions.

### Validation of Proteomic Data

The mRNA expression of three randomly selected genes (*2,3-bisphosphoglycerate-independent phosphoglycerate mutase*, *hexokinase*, and *glutathione reductase*) was evaluated in control and EI leaves (**Supplementary Figure S6**). The proteomic profiles were confirmed for the three tested genes, even though the ratio between conditions detected at the mRNA and protein levels was different, probably due to regulation at the post-transcriptional level.

## Discussion

In our first screening of rice responses to *S. oryzae* infestation using seven different cultivars, it was clearly shown that none of these cultivars present a classical resistance response, due to the rapid and somewhat similar infestation kinetics throughout the analyzed period (**Supplementary Figure S2**). Even though, physiological analysis and agronomical parameters showed that two cultivars (Puitá INTA-CL and IRGA 423) present different responses to *S. oryzae* infestation (**Figures 1–7**), being considered susceptible and tolerant, respectively. In fact, there is no consensus about the requirement for a trait be considered as a plant defense mechanism (Karban, 2011; Poelman, 2015), and most plant defenses are still characterized by proximate variables such as herbivore performance or plant damage (Wetzel et al., 2016; Erb, 2018). However, plant defenses can be surely defined as traits that reduce the negative impact of herbivores on plant reproductive success or that increase plant fitness (Erb, 2018). Tolerance mechanisms allow the plants to withstand pest injury and produce acceptable yields, maintaining the fitness under stressful conditions (Peterson et al., 2017; Sperotto et al., 2018b). For this reason, the fact that IRGA 423 did not decrease the yield under infested condition (compared to 62% in Puitá INTA-CL) was the main characteristic that encouraged us to define IRGA 423 as tolerant to *S. oryzae* infestation. It is important to highlight that under control condition (without infestation), the tolerant IRGA 423 cultivar presented a much lower yield than the susceptible Puitá INTA-CL (**Figure 5**). Although genetic immunity provides an economical method for the control of crop diseases, high levels of resistance/tolerance usually carry yield penalties (Brown, 2002). Reported in several crops, our understanding of the “cost of resistance/tolerance” on yield has improved in recent years, and novel breeding strategies to rapidly and efficiently select highly resistant/tolerant cultivars without yield penalties are being developed (Ning et al., 2017).

Chlorophyll fluorescence is a non-invasive tool commonly used for determining the behavior of the photosynthetic apparatus of control and abiotic stressed plants (Gururani et al., 2015; Rapacz et al., 2015). Our group previously detected a reduction in Pi_ABS_, S_m_, and N parameters (related to the donor and acceptor sides of PSII) in rice leaves EI by *S. oryzae* in IRGA 424 cultivar (Buffon et al., 2016). However, to the best of our knowledge, this is the first work that uses this type of photosynthetic analysis to differentiate plant susceptibility and tolerance to a biotic stress. Several parameters related to chlorophyll a fluorescence were affected in the susceptible Puitá INTA-CL cultivar during infestation, showing a worse photosynthetic performance than IRGA 423 (**Figure 2**). Puitá INTA-CL increased its energy dissipation per reaction center (DI_0_/RC – **Figure 2l**) and decreased energy flow in PSII at the four OJIP-curve/times (**Figures 2A–D**), fluorescence intensity at F300 (**Figure 2G**), energy dissipation per active leaf area (DI_0_/CS_0_ – **Figure 2M**), and light energy capture per active leaf area (TR_0_/CS_0_ – **Figure 2J**), the last one probably linked to enhanced cell death in their leaves (**Figure 6A**). Zhang et al. (2013) demonstrated that excess Ca^2+^ increased the toxicity of Hg^2+^ to PSII of cyanobacterium *Synechocystis* sp. through the increase of energy flux dissipation per reaction center (DI_0_/RC), leading to dysfunction of PSII. Rapacz et al. (2015) showed that DI_0_/RC parameter increases with increasing levels of PSII damage in wheat under low temperature. The I and P fluorescence intensities on the OJIP induction curve and the F300 parameter also decrease in wheat plants exposed to Pb stress (Kalaji and Loboda, 2007). Interestingly, tall fescue *Festuca arundinacea* leaves show a great decrease at all steps of OJIP and F300 parameters in response to high-temperature stress, but pre-acclimation treatment inhibit such declines (Hu et al., 2015). Intriguingly, Rapacz et al. (2015) suggest that DI_0_/CS_0_ values increase with increasing levels of PSII damage, which is the opposite to what we found (**Figure 2M**). More studies are needed to clarify the impact of DI_0_/CS_0_ in rice photosynthetic performance. In common wheat, TR_0_/CS_0_ values correlate well with plant survival after freezing, being an excellent indicator for prediction of winter field survival or estimation of freezing tolerance (Rapacz et al., 2015). According to Gururani et al. (2015) and Rapacz et al. (2015), the OJIP test is a reliable indicator of cold tolerance in the turfgrass *Zoysia japonica* and freezing tolerance in wheat, respectively. Therefore, we suggest for the first time that rice tolerance to *S. oryzae* (and probably to other herbivores) can also be estimated by OJIP test. According to Peterson et al. (2017), increased net photosynthetic rate after herbivory is one of the general physiological mechanisms involved in plant tolerance. The differences in photosynthetic performance presented by the susceptible Puitá INTA-CL and tolerant IRGA 423 cultivars are supported by decreased and increased numbers of photosynthesis-related proteins detected in response to *S. oryzae* infestation, respectively (**Supplementary Tables S2**, **S3**).

As a result of impaired chlorophyll a fluorescence in infested leaves of Puitá INTA-CL, an earlier senescence process was established in their leaves upon *S. oryzae* infestation (**Figure 3**). Leaf senescence is a natural and important developmental process, responsible for great part of the nitrogen mobilized to the seeds. Late senescence, which means a prolonged and maximum period of photosynthetic activity, should lead to higher yields (Jagadish et al., 2015; Diaz-Mendoza et al., 2016). However, senescence processes are also closely linked to stress conditions, which commonly anticipate this process (Wojciechowska et al., 2017). Therefore, manipulation of senescence events can be a rationale way to obtain higher yield and quality of grains (Egli, 2011; Jagadish et al., 2015). We believe that the late senescence process detected on the leaves of tolerant IRGA 423 cultivar is at least partially responsible for the better seed characteristics presented by this cultivar under *S. oryzae* infestation (**Figure 4**), including the absence of yield loss. Yet, infested leaves of susceptible Puitá INTA-CL cultivar express atATG18b protein (**Supplementary Table S5**), which is required for the formation of autophagosomes during nutrient stress and senescence in Arabidopsis (Xiong et al., 2005).

Even though we detected a lower level of H_2_O_2_ accumulation in infested leaves of the tolerant IRGA 423 cultivar (**Figure 6A**), we were not able to find a clear difference in oxidative stress-related proteins identified on the both cultivars under infested condition (**Supplementary Tables S2**, **S3**, **S5**), except a peroxidase protein 2.6-fold more abundant in tolerant IRGA 423 infested leaves (**Supplementary Table S5**). However, infested leaves of the tolerant cultivar accumulate higher levels of phenolic compounds than the susceptible one (**Figure 6B**). In plants, it is well established that phenolics can act as antioxidants by donating electrons to guaiacol-type peroxidases for the detoxification of H_2_O_2_ produced under different stress conditions, including biotic ones (Sakihama et al., 2002; Shalaby and Horwitz, 2015; Hung, 2016). Also, many structurally different phenolics rapidly accumulate to higher levels as components of an induced defense arsenal against herbivore attack (Gaquerel et al., 2014; Karabourniotis et al., 2014). For example, the larval development of the pea aphid is longer, the reproduction period is shorter, the fecundity is decreased, and the aphid population is reduced on alfalfa lines containing high levels of phenolics (Goławska and Łukasik, 2009). Therefore, we believe that part of the tolerance mechanism to *S. oryzae* infestation by IRGA 423 cultivar is due to phenolics accumulation in their leaves. Interestingly, the accumulation of phenolic compounds along with enhancement of phenylpropanoid metabolism has been observed under different environmental stress conditions (Michalak, 2006). Phenylpropanoid metabolic pathway synthesizes flavonoids, which have many diverse functions, including plant responses to stress conditions (as cold and drought – Schulz et al., 2016; Shojaie et al., 2016) and defense (Buer et al., 2010), functioning as powerful antioxidants. Under infested condition, we detected flavonol-3-*O*-glycoside-7-*O*-glucosyltransferase 1 protein, involved with flavonol biosynthesis (Sun et al., 2016), 3.6-fold more abundant on the tolerant IRGA 423 cultivar than the susceptible one (**Supplementary Table S5**), suggesting that flavonoids can also contribute to tolerate *S. oryzae* infestation.

The beneficial effects of silicon (Si) on plant resistance against biotic stresses, including insect herbivory, have been well documented in rice plants, showing positive correlations between increased Si content and enhanced insect resistance (Sidhu et al., 2013; Ye et al., 2013; Han et al., 2016). We detected higher number of amorphous silica cells and higher accumulation of SiO_2_ in infested leaves of the tolerant IRGA 423 cultivar when compared to the susceptible one (**Figure 7**). Based on these data, we strongly suggest that enhanced Si levels can also contribute to the more effective defense of IRGA 423 cultivar against *S. oryzae* mite infestation. According to Ye et al. (2013), there is a strong interaction between Si and JA in rice defense against insect herbivores involving priming of JA-mediated defense responses by Si and the promotion of Si accumulation by JA. This is reinforced by the higher expression of jasmonate *O*-methyltransferase protein on the infested leaves of tolerant IRGA 423 cultivar (**Supplementary Table S5**). Such enzyme catalyzes the methylation of JA into Me-JA, which controls plant defenses against herbivore attack (Qi et al., 2016; Huang et al., 2017). On the other hand, we detected higher expression of a GA receptor protein on the infested leaves of susceptible Puitá INTA-CL cultivar (**Supplementary Table S5**). GA regulates many essential plant developmental processes, including growth (Hou et al., 2013). As GA and JA antagonize each other in regulating plant growth and defense (Chaiwanon et al., 2016; Ning et al., 2017), we suggest that under infested condition, the susceptible Puitá INTA-CL cultivar prioritize growth over defense (showing a significant yield penalty), while the tolerant IRGA 423 prioritize defense over growth (showing no yield penalty), and this difference might contribute to the *S. oryzae* susceptiblity or tolerance, as hypothetically occurs with wild *Oryza* species of different heights (Sperotto et al., 2018a).

**FIGURE 8:**
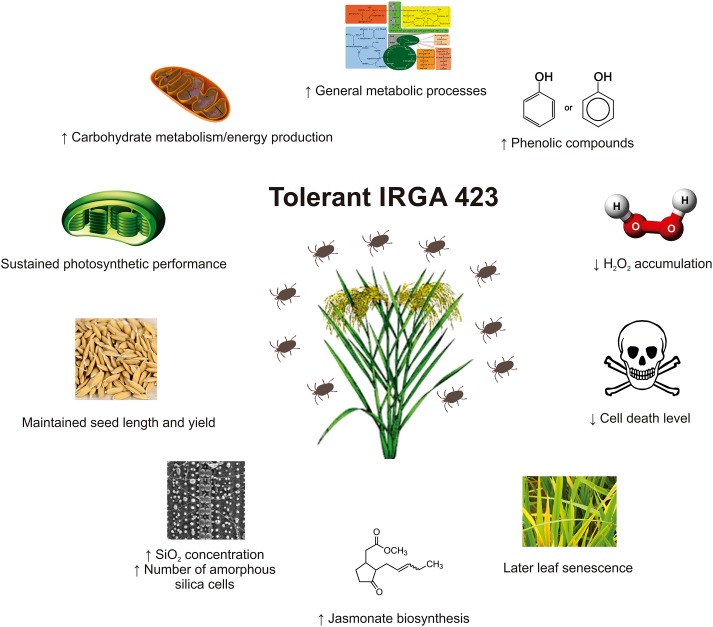
Rice mechanisms employed by IRGA 423 cultivar to tolerate *Schizotetranychus oryzae* infestation.

Physiological analyses showed that the tolerant IRGA 423 cultivar under infested condition presents better photosynthetic performance, later leaf senescence period, less affected seeds and lower yield loss, lower levels of H_2_O_2_ accumulation and cell death, higher levels of phenolic compounds (and probably flavonoids), higher level of SiO_2_ concentration in leaves, and higher number of amorphous silica cells on the leaf surface than the susceptible Puitá INTA-CL cultivar. Proteomic analysis showed that the tolerant IRGA 423 cultivar under infested condition presents a more complex and efficient response to *S. oryzae* infestation, with carbohydrate metabolism/energy production, general metabolic processes, and JA biosynthesis more active than the susceptible Puitá INTA-CL cultivar. The model in **Figure 8** summarizes the rice tolerance mechanisms employed by IRGA 423 cultivar.

## Conclusion

This is the first report evaluating the defense responses of two contrasting rice cultivars to *S. oryzae* mite infestation. Even though we were not able to find obvious signs of plant resistance in any of the tested cultivars, infested condition did not affect the number of seeds, percentage of full seeds, weight of 1,000 full seeds, seed length, and ultimatelly seed weight per plant (estimate of yield) of IRGA 423 cultivar. Therefore, this cultivar can be characterized as tolerant to *S. oryzae* infestation. Altogether, our findings are helpful to reveal the different mechanisms involved in the rice response to *S. oryzae* infestation, and could be used in future breeding programs or genetic engineering attempts aiming to increase mite tolerance in rice plants.

## Author Contributions

JS, FR, and RS conceived and designed the research. GB, EB, AR, TL, RG, JA, and AH conducted the experiments. VS and ML contributed with analytical tools. GB, EB, JA, AH, VS, and RS analyzed the data. GB and RS wrote the manuscript. All authors read and approved the manuscript.

## Conflict of Interest Statement

The authors declare that the research was conducted in the absence of any commercial or financial relationships that could be construed as a potential conflict of interest.

## Footnotes

1 https://phytozome.jgi.doe.gov/

2 https://www.blast2go.com

## Supplementary Material

FIGURE S1 Classification of infestation levels according to visual characteristics of leaves.

FIGURE S2 Pattern of infestation kinetics based on leaf damage after 5 weeks in the seven tested cultivars.

FIGURE S3 Venn diagram showing the overlap of rice proteins identified in control and early infested (EI) leaves of susceptible Puitá INTA-CL and tolerant IRGA 423 cultivars. **(a)** Puitá INTA-CL (control × infested); **(b)** IRGA 423 (control × infested); **(c)** control condition (Puitá INTA-CL × IRGA 423); **(d)** infested condition (Puitá INTA-CL × IRGA 423). In **(a)** and **(b)** dark green circles, control leaves; yellow circles, infested leaves. In **(c)** and **(d)** dark green circles, Puitá INTA-CL; yellow circles, IRGA 423. Light green means overlap in **(a)**–**(d)**.

FIGURE S4 Gene ontology annotation. Biological processes of differentially abundant and unique proteins obtained in control and EI leaves from susceptible Puitá INTA-CL and tolerant IRGA 423 cultivars.

FIGURE S5 Gene ontology annotation. Molecular functions of differentially abundant and unique proteins obtained in control and EI leaves from susceptible Puitá INTA-CL and tolerant IRGA 423 cultivars.

FIGURE S6 Relative expression levels (RT-qPCR, relative to *OsUBQ5* expression) of three randomly selected genes **(a)** *2,3-bisphosphoglycerate-independent phosphoglycerate mutase*, **(b)** *hexokinase*, **(c)** *glutathione reductase*, for which the encoded proteins were identified by proteomics as differentially abundant between the control and EI leaves from Puitá INTA-CL and IRGA 423 cultivars. Represented values are the averages of three samples ± SE. Different letters indicate that the means are different by the Tukey’s HSD test (*P* ≤ 0.05).

TABLE S1 Gene-specific PCR primers used for RT-qPCR analyses.

TABLE S2 Differentially abundant proteins in susceptible Puitá INTA-CL cultivar (control × infested condition).

TABLE S3 Differentially abundant proteins in tolerant IRGA 423 cultivar (control × infested condition).

TABLE S4 Differentially abundant proteins in control condition (susceptible Puitá INTA-CL × tolerant IRGA 423).

TABLE S5 Differentially abundant proteins in infested condition (susceptible Puitá INTA-CL × tolerant IRGA 423).

## Abbreviations

EI: early infestation
II: intermediate infestation
JA: jasmonate
LI: late infestation
PSII: photosystem II
SEM: scanning electron microscopy
