# Supplementary figures and images for: Unraveling Rice Tolerance Mechanisms Against Schizotetranychus oryzae Mite Infestation
Source: Front Plant Sci. 2018 Sep 18;9:1341. doi: 10.3389/fpls.2018.01341 (PMC6153315; doi:10.3389/fpls.2018.01341)

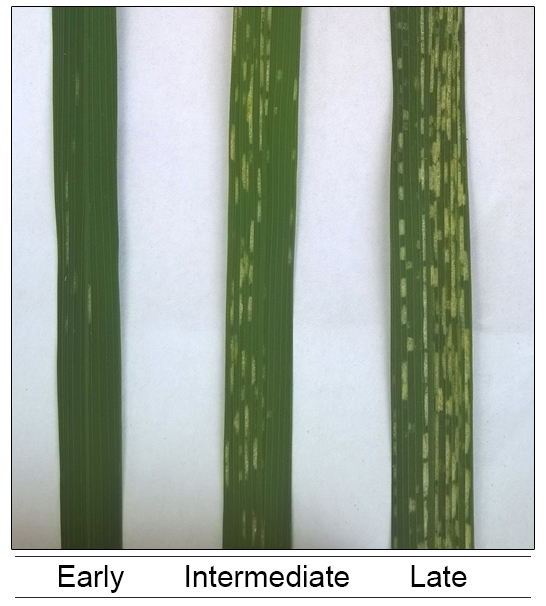

Supplement: FIGURE S1 — Classification of infestation levels according to visual characteristics of leaves. [file Image_1.JPEG]

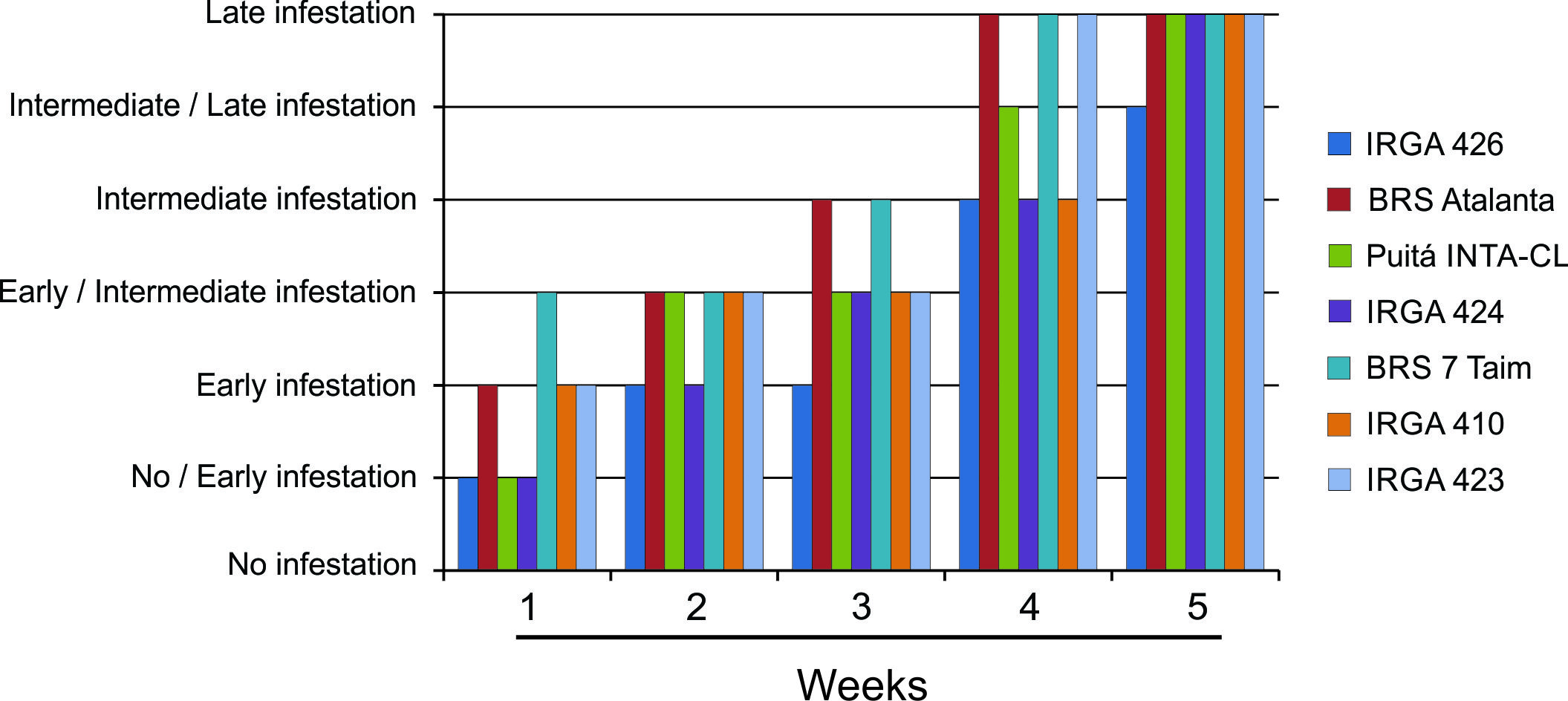

Supplement: FIGURE S2 — Pattern of infestation kinetics based on leaf damage after 5 weeks in the seven tested cultivars. [file Image_2.JPEG]

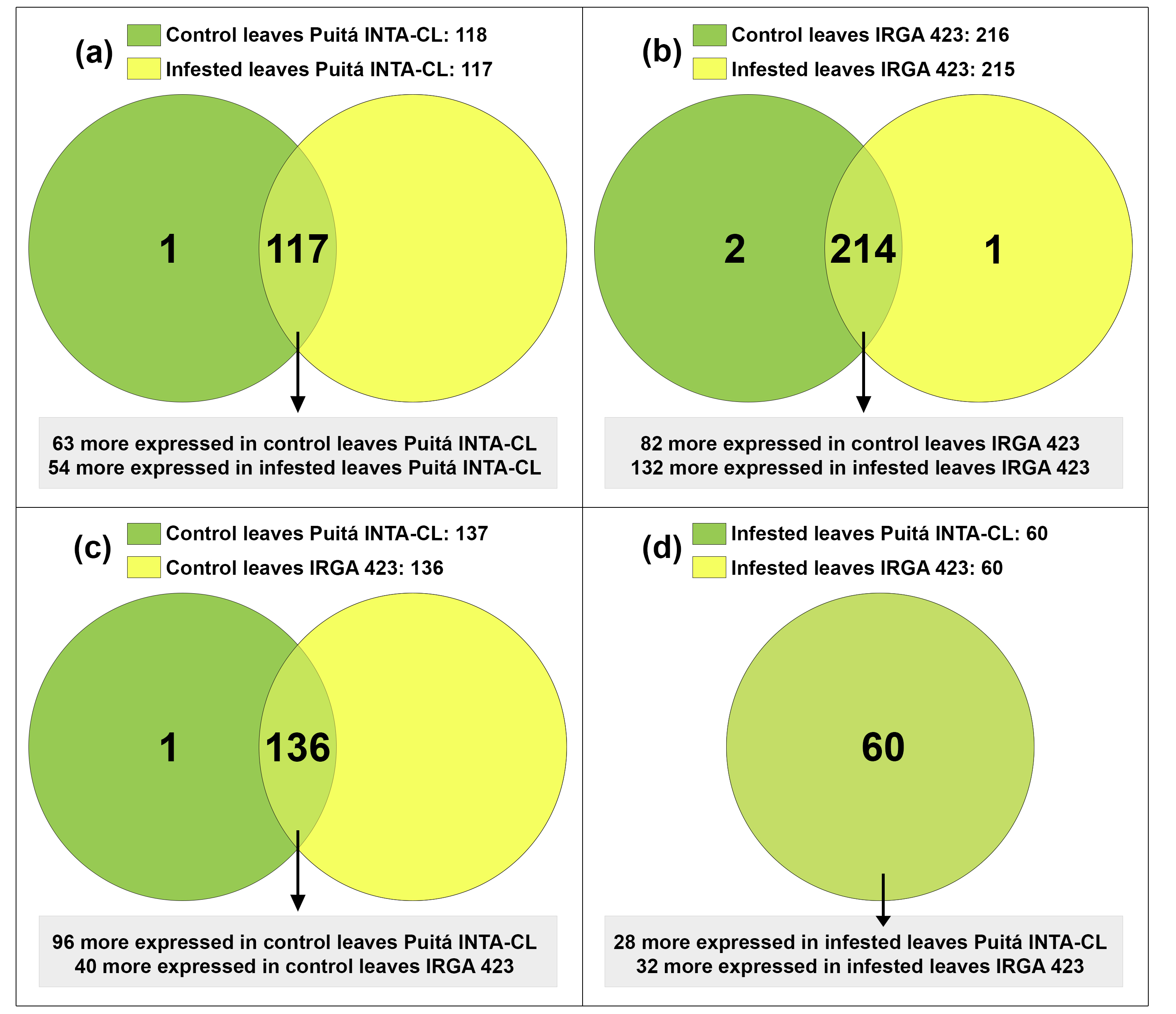

Supplement: FIGURE S3 — Venn diagram showing the overlap of rice proteins identified in control and early infested (EI) leaves of susceptible Puitá INTA-CL and tolerant IRGA 423 cultivars. (a) Puitá INTA-CL (control × infested); (b) IRGA 423 (control × infested); (c) control condition (Puitá INTA-CL × IRGA 423); (d) infested condition (Puitá INTA-CL × IRGA 423). In (a) and (b) dark green circles, control leaves; yellow circles, infested leaves. In (c) and (d) dark green circles, Puitá INTA-CL; yellow circles, IRGA 423. Light green means overlap in (a)–(d). [file Image_3.JPEG]

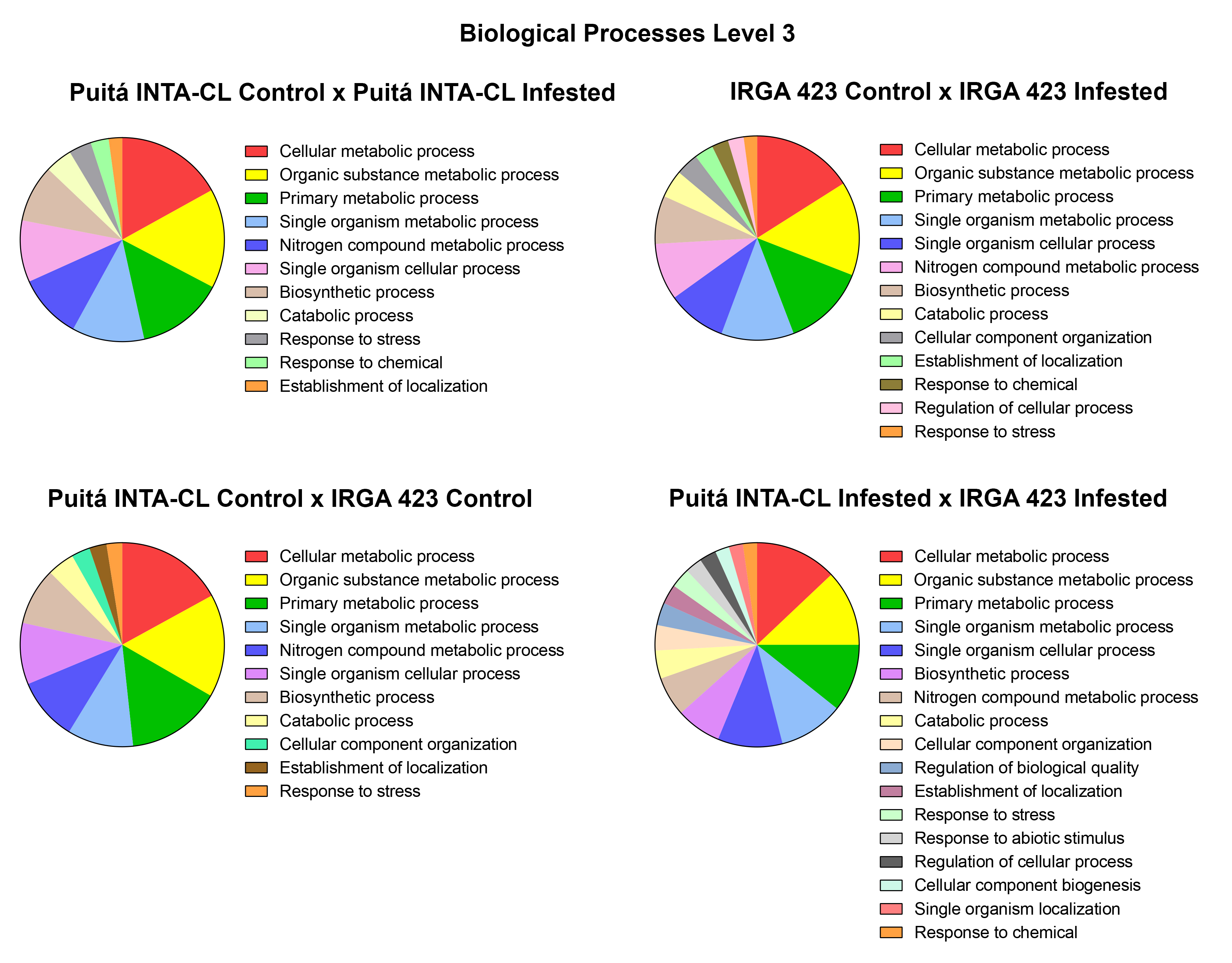

Supplement: FIGURE S4 — Gene ontology annotation. Biological processes of differentially abundant and unique proteins obtained in control and EI leaves from susceptible Puitá INTA-CL and tolerant IRGA 423 cultivars. [file Image_4.JPEG]

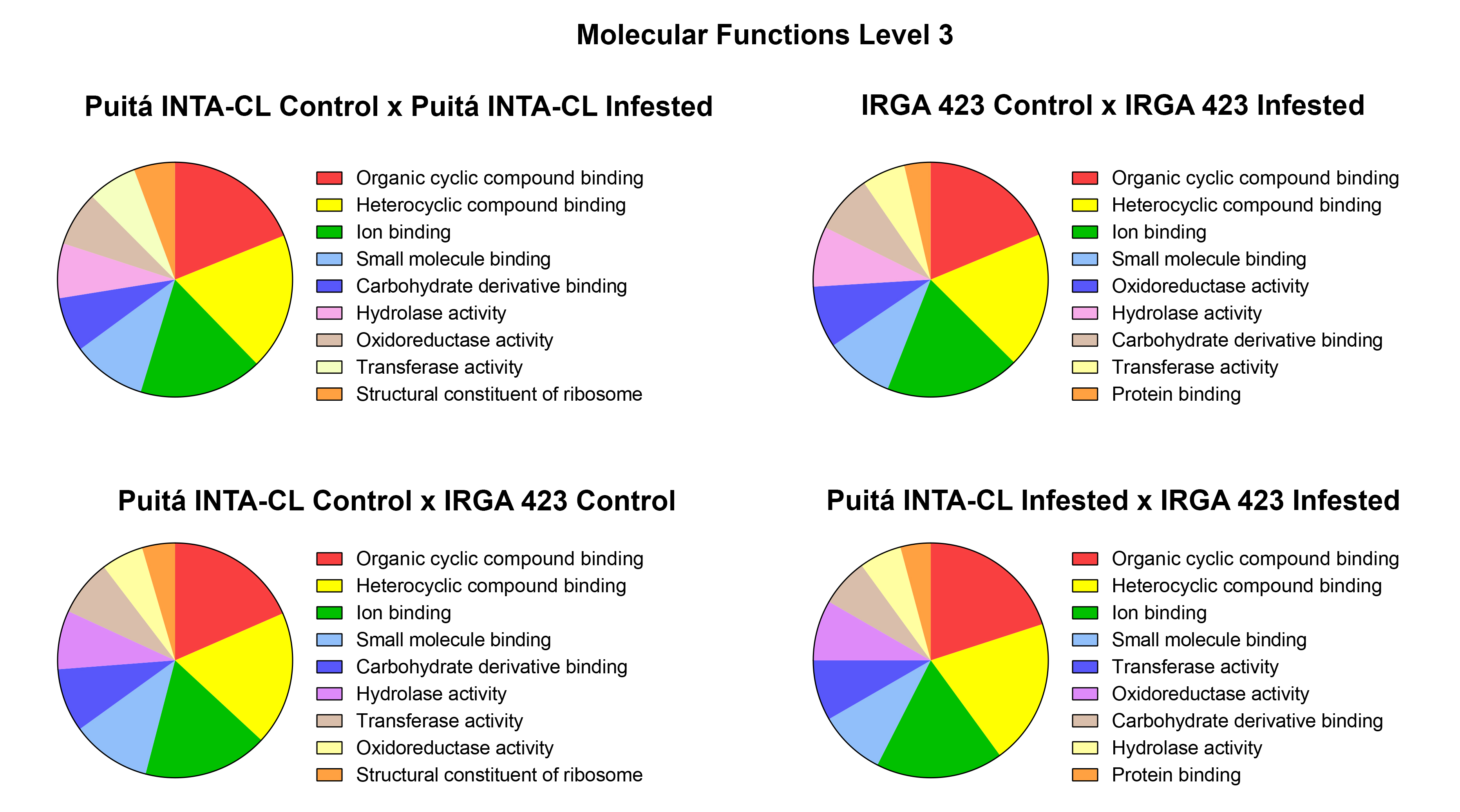

Supplement: FIGURE S5 — Gene ontology annotation. Molecular functions of differentially abundant and unique proteins obtained in control and EI leaves from susceptible Puitá INTA-CL and tolerant IRGA 423 cultivars. [file Image_5.JPEG]

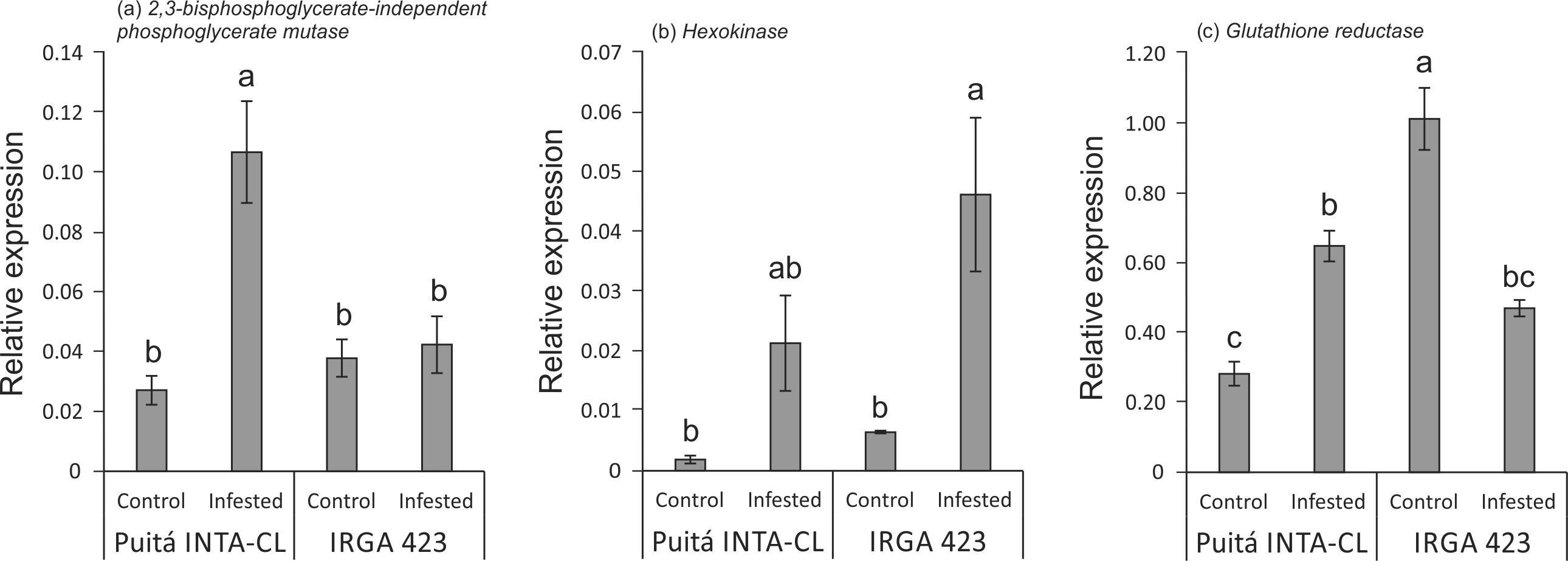

Supplement: FIGURE S6 — Relative expression levels (RT-qPCR, relative to OsUBQ5 expression) of three randomly selected genes (a) 2,3-bisphosphoglycerate-independent phosphoglycerate mutase, (b) hexokinase, (c) glutathione reductase, for which the encoded proteins were identified by proteomics as differentially abundant between the control and EI leaves from Puitá INTA-CL and IRGA 423 cultivars. Represented values are the averages of three samples ± SE. Different letters indicate that the means are different by the Tukey’s HSD test (P ≤ 0.05). [file Image_6.JPEG]
