# Supplementary material for: Unraveling Rice Tolerance Mechanisms Against Schizotetranychus oryzae Mite Infestation
Source: Front Plant Sci. 2018 Sep 18;9:1341. doi: 10.3389/fpls.2018.01341 (PMC6153315; doi:10.3389/fpls.2018.01341)
Supplement: TABLE S1 — Gene-specific PCR primers used for RT-qPCR analyses. [file Table_1.DOCX]

| **Supplementary Table 1. Gene-specific PCR primers used for RT-qPCR analyses.** | |  |
| --- | --- | --- |
|  |  |  |
| **Gene** | **Forward primer 5’ → 3’** | **Reverse primer 5’ → 3’** |
| *2,3-bisphosphoglycerate-independent phosphoglycerate mutase* | TGGTCTCTGCGATGTTGAAT | TCACAACATCACGATGCAAA |
| *Hexokinase* | GCATTTGAGGAAAAGAAACACC | GGCCACACAAAATTGAGGAT |
| *Glutathione reductase* | ATAGCGCTGACTGGCATTCT | ACAAAATTTCCAGGCTGTGG |
| *OsSGR* | CTACCAAACCGAGCCAAAAT | ACCAAAACGACTCTTGACAGC |
