# Supplementary material for: Unraveling Rice Tolerance Mechanisms Against Schizotetranychus oryzae Mite Infestation
Source: Front Plant Sci. 2018 Sep 18;9:1341. doi: 10.3389/fpls.2018.01341 (PMC6153315; doi:10.3389/fpls.2018.01341)
Supplement: TABLE S2 — Differentially abundant proteins in susceptible Puitá INTA-CL cultivar (control × infested condition). [file Table_2.DOCX]

| **Puitá INTA-CL Control x Infested - Proteins unique or more expressed in control leaves** | | | | | | | | | |  |
| --- | --- | --- | --- | --- | --- | --- | --- | --- | --- | --- |
| **Functional categories** | **Description** | | **Locus** | | **ANOVA** | | **Fold change Infested** **/Control** | **Unique to Control** | |  |
| Translation-related | tRNA synthetases class II domain containing protein | | LOC_Os01g27520 | | 0.01247 | | 0.62837 |  | |  |
|  | nascent polypeptide-associated complex subunit alpha | | LOC_Os03g02960 | | 0.02517 | | 0.50108 |  | |  |
|  | elongation factor protein | | LOC_Os07g46750 | | 0.00012 | | 0.66317 |  | |  |
|  | 40S ribosomal protein S3-1 | | LOC_Os03g38000 | | 0.01404 | | 0.65389 |  | |  |
|  | ribosomal L18p/L5e family protein | | LOC_Os03g61260 | | 0.01139 | | 0.63498 |  | |  |
|  | 60S ribosomal protein L8 | | LOC_Os12g38000 | | 0.02130 | | 0.62877 |  | |  |
|  | ribosomal protein L7/L12 C-terminal domain containing protein | | LOC_Os01g47330 | | 0.02464 | | 0.54914 |  | |  |
|  | L11 domain containing ribosomal protein | | LOC_Os03g03020 | | 0.00888 | | 0.53995 |  | |  |
|  | ribosomal protein L5 | | LOC_Os03g03360 | | 0.00069 | | 0.52510 |  | |  |
|  | L11 domain containing ribosomal protein | | LOC_Os04g50990 | | 0.00690 | | 0.49737 |  | |  |
|  | 60S acidic ribosomal protein | | LOC_Os08g02340 | | 0.00386 | | 0.49457 |  | |  |
|  | plastid-specific 30S ribosomal protein 1 | | LOC_Os03g63950 | | 0.00495 | | 0.47812 |  | |  |
|  | 60S acidic ribosomal protein | | LOC_Os05g37330 | | 0.03947 | | 0.47565 |  | |  |
|  | 40S ribosomal protein S27 | | LOC_Os04g27860 | | 0.00228 | | 0.44781 |  | |  |
|  | chloroplast 50S ribosomal protein L16 | | LOC_Os04g16826 | | 0.00676 | | 0.43905 |  | |  |
|  | ribosomal protein L13 | | LOC_Os03g54890 | | 0.02249 | | 0.41500 |  | |  |
|  | elongation factor | | LOC_Os01g53900 | | 0.00014 | | 0.32316 |  | |  |
|  | ribosomal protein L3 | | LOC_Os12g07010 | | 0.00100 | | 0.31672 |  | |  |
|  | 60S ribosomal protein L19-3 | | LOC_Os03g21940 | | 0.02606 | | 0.27428 |  | |  |
|  | ribosomal protein L4 | | LOC_Os03g15870 | | 0.00182 | | 0.26562 |  | |  |
|  | ribosomal protein L4 | | LOC_Os03g58204 | | 0.02255 | | 0.19107 |  | |  |
| General metabolic processes | aminotransferase, class I and II | | LOC_Os10g25130 | | 0.03274 | | 0.37717 |  | |  |
|  | D-alanine--D-alanine ligase family | | LOC_Os07g49110 | | 0.00001 | | 0.56299 |  | |  |
|  | CBS domain containing membrane protein | | LOC_Os03g52690 | | 0.04317 | | 0.53289 |  | |  |
|  | CBS domain containing membrane protein | | LOC_Os02g57280 | | 0.02068 | | 0.39157 |  | |  |
|  | nucleoside diphosphate kinase | | LOC_Os05g51700 | | 0.00305 | | 0.66325 |  | |  |
|  | methylisocitrate lyase 2 | | LOC_Os04g31700 | | 0.00008 | | 0.59222 |  | |  |
|  | hydrolase. NUDIX family | | LOC_Os05g34180 | | 0.00111 | | 0.52688 |  | |  |
|  | nucleoside diphosphate kinase | | LOC_Os10g41410 | | 0.00020 | | 0.48180 |  | |  |
| Carbohydrate metabolism and energy production | glyceraldehyde-3-phosphate dehydrogenase | | LOC_Os08g03290 | | 0.00003 | | 0.52463 |  | |  |
|  | 2.3-bisphosphoglycerate-independent phosphoglycerate mutase | | LOC_Os01g60190 | | 0.00343 | | 0.47169 |  | |  |
|  | enolase | | LOC_Os10g08550 | | 0.00774 | | 0.63004 |  | |  |
|  | **2.3-bisphosphoglycerate-independent phosphoglycerate mutase** | | **LOC_Os05g40420** | | **0.00249** | | **0.41798** |  | |  |
|  | pyruvate kinase | | LOC_Os11g05110 | | 0.00016 | | 0.15590 |  | |  |
|  | glyceraldehyde-3-phosphate dehydrogenase | | LOC_Os04g40950 | | 0.00480 | | 0.37650 |  | |  |
| Oxidative stress-related | catalase isozyme A | | LOC_Os02g02400 | | 0.00486 | | 0.64540 |  | |  |
|  | glutathione S-transferase | | LOC_Os09g29200 | | 0.00214 | | 0.46043 |  | |  |
|  | peroxidase precursor | | LOC_Os07g48030 | | 0.02891 | | 0.42510 |  | |  |
|  | **glutathione reductase** | | **LOC_Os03g06740** | | **0.03450** | | **0.37694** |  | |  |
| Photosynthesis | calvin cycle protein CP12 | | LOC_Os03g19380 | | 0.00730 | | 0.26200 |  | |  |
|  | photosystem I P700 chlorophyll a apoprotein A2 | | LOC_Os10g21248 | | 0.02314 | | 0.65287 |  | |  |
|  | PsbP | | LOC_Os08g25900 | | 0.00363 | | 0.55306 |  | |  |
|  | rhodanese-like domain containing protein | | LOC_Os09g36040 | | 0.01170 | | 0.29527 |  | |  |
| Protein modification/degradation | LTPL7 - Protease inhibitor/seed storage/LTP family protein | | LOC_Os11g02369 | | 0.04473 | | 0.48885 |  | |  |
|  | kinase. pfkB family | | LOC_Os08g02120 | | 0.00071 | | 0.43862 |  | |  |
|  | DnaK family protein | | LOC_Os05g38530 | | 0.02357 | | 0.63567 |  | |  |
|  | serine/threonine protein phosphatase 2A 55 kDa regulatory subunit B | | LOC_Os02g40454 | | 0.00261 | | 0.10963 |  | |  |
| Stress response | NBS-LRR disease resistance protein | | LOC_Os12g28070 | | 0.00306 | | 0.66484 |  | |  |
|  | pathogenesis-related Bet v I family protein | | LOC_Os12g36880 | | 0.02579 | | 0.59464 |  | |  |
|  | heat shock protein | | LOC_Os08g39140 | | 0.04274 | | 0.50045 |  | |  |
|  | NBS-LRR type disease resistance protein Rps1-k-2 | | LOC_Os12g10180 | | 0.01311 | | 0.29457 |  | |  |
| Secondary metabolism | chalcone synthase | | LOC_Os01g41834 | | 0.04382 | | 0.32448 |  | |  |
|  | dirigent | | LOC_Os11g42550 | | 0.03714 | | 0.65609 |  | |  |
| Lipid metabolism | 3-ketoacyl-CoA thiolase | | LOC_Os02g57260 | | 0.00192 | | 0.57371 |  | |  |
| Transport-related | outer mitochondrial membrane porin | | LOC_Os05g45950 | | 0.00057 | | 0.55221 |  | |  |
| Ca^2+^-signaling-related | OsCam2 - Calmodulin | | LOC_Os05g41210 | | 0.01310 | | 0.38714 |  | |  |
| Cell structure and cell division | WD domain. G-beta repeat domain containing protein | | LOC_Os05g47890 | | 0.04511 | | 0.33116 |  | |  |
| Transcription-related | KH domain containing protein | | LOC_Os03g60110 | | 0.00003 | | - | x | |  |
| Others | actin | | LOC_Os01g73310 | | 0.00130 | | 0.36018 |  | |  |
|  | ribonuclease T2 family domain containing protein | | LOC_Os09g36680 | | 0.00382 | | 0.44303 |  | |  |
|  | ribonuclease T2 family domain containing protein | | LOC_Os09g36700 | | 0.00937 | | 0.28801 |  | |  |
| Unknown | expressed protein | | ChrSy.fgenesh.mR | | 0.04253 | | 0.63727 |  | |  |
|  | expressed protein | | LOC_Os04g01540 | | 0.00044 | | 0.38269 |  | |  |
|  | protein of unknown function domain containing protein | | LOC_Os01g07810 | | 0.01254 | | 0.34425 |  | |  |
| **Puitá INTA-CL Control x Infested - Proteins more expressed in infested leaves** | | | | | | | | | | |
| **Functional categories** | | **Description** | | **Locus** | | **ANOVA** | | **Fold change Infested** **/Control** |  | |
| General metabolic processes | | receptor-like protein kinase 2 precursor | | LOC_Os02g06280 | | 0.00154 | | 14.23607 |  | |
|  |  | arginase | | LOC_Os04g01590 | | 0.00225 | | 8.82457 |  | |
|  |  | SOR/SNZ family protein | | LOC_Os10g01080 | | 0.00136 | | 2.30789 |  | |
|  |  | transferase family protein | | LOC_Os08g01980 | | 0.00471 | | 2.24550 |  | |
|  |  | amine oxidase precursor | | LOC_Os04g20164 | | 0.00476 | | 2.12670 |  | |
|  |  | SOR/SNZ family protein | | LOC_Os07g01020 | | 0.01040 | | 1.66365 |  | |
|  |  | inositol-1-monophosphatase | | LOC_Os02g07350 | | 0.01477 | | 1.58857 |  | |
|  |  | dehydrogenase | | LOC_Os09g23540 | | 0.01070 | | 1.54536 |  | |
|  |  | cbbY | | LOC_Os03g36750 | | 0.01413 | | 1.52857 |  | |
| Oxidative stress-related | | glyoxalase family protein | | LOC_Os08g09250 | | 0.01915 | | 3.33447 |  | |
|  |  | rubredoxin family protein | | LOC_Os08g23410 | | 0.00126 | | 2.57552 |  | |
|  |  | oxidoreductase. aldo/keto reductase family protein | | LOC_Os05g38230 | | 0.01984 | | 2.19904 |  | |
|  |  | copper/zinc superoxide dismutase | | LOC_Os08g44770 | | 0.00005 | | 2.15070 |  | |
|  |  | peroxidase precursor | | LOC_Os03g22010 | | 0.00493 | | 1.95639 |  | |
|  |  | peroxidase precursor | | LOC_Os09g29490 | | 0.03425 | | 1.82005 |  | |
|  |  | thioredoxin | | LOC_Os01g68480 | | 0.00177 | | 1.76178 |  | |
|  |  | glutathione S-transferase | | LOC_Os03g04260 | | 0.00218 | | 1.69288 |  | |
|  |  | thioredoxin | | LOC_Os07g29410 | | 0.00548 | | 1.66243 |  | |
|  |  | oxidoreductase. aldo/keto reductase family protein | | LOC_Os07g05000 | | 0.00135 | | 1.59536 |  | |
| Protein modification/degradation | | chaperone protein clpB 1 | | LOC_Os02g08490 | | 0.00252 | | 2.71876 |  | |
|  |  | LTPL113 - Protease inhibitor/seed storage/LTP family protein precursor | | LOC_Os02g44320 | | 0.01122 | | 2.07284 |  | |
|  |  | OsFtsH4 FtsH protease, homologue to AtFtsH4 | | LOC_Os01g39250 | | 0.02095 | | 1.52309 |  | |
|  |  | peptidyl-prolyl cis-trans isomerase FKBP-type | | LOC_Os06g45340 | | 0.01153 | | 1.64891 |  | |
|  |  | oryzain alpha chain precursor | | LOC_Os04g55650 | | 0.00013 | | 1.64419 |  | |
| Carbohydrate metabolism and energy production | | ATP synthase delta chain | | LOC_Os07g31300 | | 0.02523 | | 4.97600 |  | |
|  |  | ATP synthase B chain | | LOC_Os03g17070 | | 0.00840 | | 1.98271 |  | |
|  |  | ATP synthase subunit beta | | LOC_Os06g39740 | | 0.02309 | | 1.70922 |  | |
|  |  | **hexokinase** | | **LOC_Os07g09890** | | **0.00273** | | **1.62836** |  | |
|  |  | fructose-1,6-bisphosphatase | | LOC_Os03g16050 | | 0.01582 | | 1.52932 |  | |
| Lipid metabolism | | enoyl-acyl-carrier-protein reductase NADH | | LOC_Os08g23810 | | 0.00825 | | 2.46474 |  | |
|  |  | phospholipase D | | LOC_Os01g07760 | | 0.00196 | | 1.52010 |  | |
| Amino acid metabolism | | ketol-acid reductoisomerase | | LOC_Os01g46380 | | 0.04536 | | 1.64203 |  | |
|  |  | peptide methionine sulfoxide reductase | | LOC_Os10g41400 | | 0.00132 | | 1.61400 |  | |
| Photosynthesis | | photosystem II 10 kDa polypeptide | | LOC_Os08g10020 | | 0.02012 | | 1.65541 |  | |
|  |  | phosphoenolpyruvate carboxylase | | LOC_Os09g14670 | | 0.01228 | | 1.54771 |  | |
| Stress response | | stress responsive protein | | LOC_Os01g01450 | | 0.00400 | | 2.92562 |  | |
|  |  | thaumatin family domain containing protein | | LOC_Os12g38120 | | 0.00530 | | 2.03927 |  | |
| Cell structure and cell division | | myosin-Vb | | LOC_Os07g37560 | | 0.00127 | | 3.37962 |  | |
| Hormone-related | | gibberellin receptor | | LOC_Os09g28630 | | 0.00519 | | 2.46253 |  | |
| Translation-related | | G-patch domain containing protein | | LOC_Os03g14860 | | 0.00589 | | 2.06331 |  | |
| Transport-related | | OsCHL Chloroplastic lipocalin | | LOC_Os04g53490 | | 0.00458 | | 1.67202 |  | |
| Transcription-related | | RNA recognition motif containing protein | | LOC_Os03g46770 | | 0.04808 | | 1.54432 |  | |
| Cell structure and cell division | | kinesin motor domain containing protein | | LOC_Os04g53760 | | 0.01765 | | 1.53000 |  | |
| Others | | C2 domain containing protein | | LOC_Os02g58230 | | 0.00477 | | 1.90617 |  | |
|  |  | actin | | LOC_Os01g64630 | | 0.01174 | | 1.68875 |  | |
|  |  | dienelactone hydrolase family protein | | LOC_Os01g34700 | | 0.01290 | | 1.76207 |  | |
|  |  | membrane-associated 30 kDa protein | | LOC_Os01g67000 | | 0.01878 | | 1.52919 |  | |
| Unknown | | expressed protein | | LOC_Os03g61090 | | 0.00987 | | 2.13356 |  | |
|  |  | expressed protein | | LOC_Os02g28680 | | 0.01691 | | 1.85233 |  | |
|  |  | expressed protein | | LOC_Os07g10620 | | 0.00381 | | 1.83884 |  | |
|  |  | hypothetical protein | | LOC_Os12g13530 | | 0.01766 | | 1.70852 |  | |
|  |  | expressed protein | | LOC_Os08g16570 | | 0.00028 | | 1.53359 |  | |
|  |  | expressed protein | | LOC_Os02g48480 | | 0.04198 | | 1.52532 |  | |
|  |  | expressed protein | | LOC_Os07g09800 | | 0.00538 | | 1.52098 |  | |

**Obs: Bold and underlined sequences were confirmed by RT-qPCR**.
