# Supplementary material for: Unraveling Rice Tolerance Mechanisms Against Schizotetranychus oryzae Mite Infestation
Source: Front Plant Sci. 2018 Sep 18;9:1341. doi: 10.3389/fpls.2018.01341 (PMC6153315; doi:10.3389/fpls.2018.01341)
Supplement: TABLE S3 — Differentially abundant proteins in tolerant IRGA 423 cultivar (control × infested condition). [file Table_3.DOCX]

| **IRGA 423 Control x Infested - Proteins unique or more expressed in control leaves** | | | | | | | | | | | |
| --- | --- | --- | --- | --- | --- | --- | --- | --- | --- | --- | --- |
| **Functional categories** | | **Description** | **Locus** | | **ANOVA** | | | **Fold change Infested** **/Control** | | **Unique to Control** | |
| Carbohydrate metabolism and energy production | | NAD dependent epimerase/dehydratase | LOC_Os10g28200 | | 0.00113 | | | 0.65596 | |  | |
|  |  | fructose-bisphospate aldolase isozyme | LOC_Os05g33380 | | 0.01488 | | | 0.58929 | |  | |
|  |  | ATP synthase | LOC_Os01g49190 | | 0.03692 | | | 0.49972 | |  | |
|  |  | NADP-dependent malic enzyme | LOC_Os01g52500 | | 0.00295 | | | 0.41873 | |  | |
|  |  | ATP synthase gamma chain | LOC_Os10g17280 | | 0.03551 | | | 0.38626 | |  | |
|  |  | AAA-type ATPase family protein | LOC_Os04g56320 | | 0.00099 | | | 0.36484 | |  | |
|  |  | 2,3-bisphosphoglycerate-independent phosphoglycerate mutase | LOC_Os01g60190 | | 0.00132 | | | 0.29229 | |  | |
|  |  | ATP synthase subunit beta | LOC_Os06g39740 | | 0.00000 | | | 0.22420 | |  | |
|  |  | pyruvate kinase | LOC_Os11g05110 | | 0.00029 | | | 0.21793 | |  | |
|  |  | NAD binding domain of 6-phosphogluconate dehydrogenase containing protein | LOC_Os01g39270 | | 0.00014 | | | 0.17711 | |  | |
|  |  | **2,3-bisphosphoglycerate-independent phosphoglycerate mutase** | **LOC_Os05g40420** | | **0.00000** | | | **0.02343** | |  | |
| Oxidative stress-related | | OsGrx_S17 - glutaredoxin subgroup II | LOC_Os10g35720 | | 0.04704 | | | 0.66137 | |  | |
|  |  | glyoxalase family protein | LOC_Os05g22970 | | 0.00364 | | | 0.64413 | |  | |
|  |  | peroxidase precursor | LOC_Os01g22352 | | 0.01694 | | | 0.62774 | |  | |
|  |  | superoxide dismutase, mitochondrial precursor | LOC_Os05g25850 | | 0.00331 | | | 0.61227 | |  | |
|  |  | thioredoxin | LOC_Os07g08840 | | 0.00769 | | | 0.58374 | |  | |
|  |  | peroxidase precursor | LOC_Os01g22336 | | 0.01452 | | | 0.23096 | |  | |
|  |  | rubredoxin family protein | LOC_Os08g23410 | | 0.00369 | | | 0.22875 | |  | |
|  |  | OsGrx_S16 - glutaredoxin subgroup II | LOC_Os12g07650 | | 0.00012 | | | 0.22260 | |  | |
|  |  | **glutathione reductase** | **LOC_Os03g06740** | | **0.00792** | | | **0.15802** | |  | |
|  |  | peroxidase precursor | LOC_Os01g22249 | | 0.00014 | | | 0.06032 | |  | |
| Translation-related | | ribosomal protein L13 | LOC_Os03g37970 | | 0.00908 | | | 0.65575 | |  | |
|  |  | 60S acidic ribosomal protein | LOC_Os05g37330 | | 0.03499 | | | 0.65314 | |  | |
|  |  | plastid-specific 30S ribosomal protein 1, chloroplast precursor | LOC_Os03g63950 | | 0.02375 | | | 0.61882 | |  | |
|  |  | ribosomal protein S17 | LOC_Os04g52361 | | 0.03672 | | | 0.58485 | |  | |
|  |  | ribosomal protein L7/L12 C-terminal domain containing protein | LOC_Os01g47330 | | 0.00115 | | | 0.51788 | |  | |
|  |  | L11 domain containing ribosomal protein | LOC_Os04g50990 | | 0.02503 | | | 0.44783 | |  | |
|  |  | ligA | LOC_Os06g15980 | | 0.00003 | | | 0.43636 | |  | |
|  |  | ribosomal L9 | LOC_Os02g57670 | | 0.04442 | | | 0.40125 | |  | |
|  |  | elongation factor protein | LOC_Os07g42300 | | 0.00101 | | | 0.37424 | |  | |
|  |  | elongation factor | LOC_Os02g32030 | | 0.00089 | | | 0.26769 | |  | |
|  |  | ribosomal protein | LOC_Os02g06700 | | 0.00550 | | | 0.07660 | |  | |
| Photosynthesis | | oxygen evolving enhancer protein 3 domain containing protein | LOC_Os07g01480 | | 0.00048 | | | 0.60845 | |  | |
|  |  | photosystem I reaction center subunit, chloroplast precursor | LOC_Os09g30340 | | 0.00047 | | | 0.59162 | |  | |
|  |  | cytochrome b6-f complex iron-sulfur subunit, chloroplast precursor | LOC_Os07g37030 | | 0.00221 | | | 0.58720 | |  | |
|  |  | ribulose bisphosphate carboxylase large chain precursor | LOC_Os12g10580 | | 0.04033 | | | 0.56542 | |  | |
|  |  | PsbP | LOC_Os03g17174 | | 0.00028 | | | 0.21764 | |  | |
|  |  | PsbP | LOC_Os08g25900 | | 0.00699 | | | 0.12223 | |  | |
| Protein modification/degradation | | peptidyl-prolyl cis-trans isomerase, FKBP-type | LOC_Os06g20320 | | 0.02719 | | | 0.61890 | |  | |
|  |  | oligopeptidase | LOC_Os02g58340 | | 0.00070 | | | 0.59228 | |  | |
|  |  | cysteine proteinase 1 precursor | LOC_Os02g27030 | | 0.00275 | | | 0.50402 | |  | |
|  |  | kinase, pfkB family | LOC_Os08g02120 | | 0.00054 | | | 0.36381 | |  | |
|  |  | cell division protease ftsH homolog 4 | LOC_Os04g39190 | | 0.00009 | | | 0.19945 | |  | |
| General metabolic processes | | 14-3-3 protein | LOC_Os02g36974 | | 0.01344 | | | 0.58269 | |  | |
|  |  | dehydrogenase | LOC_Os09g23540 | | 0.00972 | | | 0.53445 | |  | |
|  |  | hydrolase, NUDIX family | LOC_Os05g34180 | | 0.03578 | | | 0.35770 | |  | |
|  |  | kelch repeat protein | LOC_Os04g40740 | | 0.00144 | | | 0.17340 | |  | |
| Stress response | | universal stress protein domain containing protein | LOC_Os03g53900 | | 0.00819 | | | 0.37533 | |  | |
|  |  | thaumatin | LOC_Os12g43430 | | 0.01207 | | | 0.25086 | |  | |
|  |  | phosducin-like protein 3 | LOC_Os01g08960 | | 0.00033 | | | 0.22292 | |  | |
|  |  | thaumatin | LOC_Os12g43410 | | 0.01336 | | | 0.18688 | |  | |
|  |  | NBS-LRR type disease resistance protein Rps1-k-2 | LOC_Os12g10180 | | 0.00003 | | | 0.04728 | |  | |
| Transport related | | pleiotropic drug resistance protein 4 | LOC_Os09g16458 | | 0.04006 | | | 0.65529 | |  | |
|  |  | ABC transporter, ATP-binding protein | LOC_Os03g21490 | | 0.00000 | | | 0.39804 | |  | |
|  |  | outer mitochondrial membrane porin | LOC_Os01g51770 | | 0.02060 | | | 0.19700 | |  | |
|  |  | cation efflux family protein | LOC_Os04g23180 | | 0.00633 | | | 0.16475 | |  | |
| Transcription-related | | RNA recognition motif containing protein | LOC_Os07g06450 | | 0.03044 | | | 0.44026 | |  | |
|  |  | pentatricopeptide | LOC_Os12g18640 | | 0.00299 | | | 0.43793 | |  | |
|  |  | KH domain containing protein | LOC_Os03g60110 | | 0.00000 | | | - | | x | |
| Lipid metabolism | | hydroxyacid oxidase 1 | LOC_Os07g05820 | | 0.00998 | | | 0.62571 | |  | |
|  |  | non-lysosomal glucosylceramidase | LOC_Os11g13810 | | 0.00037 | | | 0.58069 | |  | |
|  |  | acyl carrier protein | LOC_Os11g31900 | | 0.01338 | | | 0.46331 | |  | |
| Cell structure and cell division | | WD domain, G-beta repeat domain containing protein | LOC_Os05g47890 | | 0.00327 | | | 0.39408 | |  | |
|  |  | tubulin/FtsZ domain containing protein | LOC_Os03g11970 | | 0.00299 | | | 0.04686 | |  | |
| Amino acid metabolism | | glutamine synthetase, catalytic | LOC_Os02g50240 | | 0.00283 | | | 0.66648 | |  | |
|  |  | cysteine synthase | LOC_Os03g53650 | | 0.00731 | | | 0.65125 | |  | |
| DNA structure maintenance | | core histone H2A/H2B/H3/H4 | LOC_Os08g33100 | | 0.00001 | | | - | | x | |
| Ca^2+^-signaling related | | OsCam2 - Calmodulin | LOC_Os05g41210 | | 0.00986 | | | 0.54128 | |  | |
| Secondary metabolism | | terpene synthase | LOC_Os04g26980 | | 0.00345 | | | 0.09545 | |  | |
| Storage related | | glutelin | LOC_Os02g25640 | | 0.00186 | | | 0.09521 | |  | |
| Others | | arsenate reductase | LOC_Os02g49680 | | 0.02751 | | | 0.65703 | |  | |
|  |  | ribonuclease T2 family domain containing protein | LOC_Os09g36680 | | 0.01101 | | | 0.39786 | |  | |
|  |  | OsMADS55 - MADS-box family gene with MIKCc type-box | LOC_Os06g11330 | | 0.00521 | | | 0.37567 | |  | |
|  |  | ribonuclease T2 family domain containing protein | LOC_Os09g36700 | | 0.03791 | | | 0.35414 | |  | |
|  |  | bundle sheath defective protein 2 | LOC_Os06g22690 | | 0.02096 | | | 0.29869 | |  | |
|  |  | sex determination protein tasselseed-2 | LOC_Os04g33240 | | 0.02704 | | | 0.28272 | |  | |
|  |  | ATP11 protein | LOC_Os02g20860 | | 0.00474 | | | 0.28146 | |  | |
|  |  | endoribonuclease | LOC_Os07g33240 | | 0.00724 | | | 0.27270 | |  | |
|  |  | homeobox protein knotted-1-like 10 | LOC_Os03g47016 | | 0.00565 | | | 0.25301 | |  | |
|  |  | metallo-beta-lactamase family protein | LOC_Os03g21460 | | 0.00070 | | | 0.06988 | |  | |
| Unknown | | expressed protein | LOC_Os04g01540 | | 0.00222 | | | 0.17765 | |  | |
|  |  | expressed protein | LOC_Os07g28610 | | 0.00871 | | | 0.58838 | |  | |
|  |  | expressed protein | LOC_Os04g57020 | | 0.00844 | | | 0.25020 | |  | |
|  |  | expressed protein | LOC_Os05g49080 | | 0.00029 | | | 0.06118 | |  | |
| **IRGA 423 Control/Infested - Proteins unique or more expressed in infested leaves** | | | | | | | | | | |  |
| **Functional categories** | **Description** | | | **Locus** | | **ANOVA** | **Fold change Infested** **/Control** | | **Unique to Infested** | |  |
| Protein modification/degradation | cysteine proteinase EP-B 1 precursor | | | LOC_Os09g39100 | | 0.00604 | 38.25650 | |  | |  |
|  | 4-nitrophenylphosphatase | | | LOC_Os09g08660 | | 0.00022 | 23.29595 | |  | |  |
|  | DnaK family protein | | | LOC_Os12g14070 | | 0.00759 | 3.71462 | |  | |  |
|  | peptidyl-prolyl cis-trans isomerase, FKBP-type | | | LOC_Os02g51570 | | 0.00167 | 3.67823 | |  | |  |
|  | peptidyl-prolyl cis-trans isomerase CYP37 | | | LOC_Os07g37830 | | 0.00017 | 2.42211 | |  | |  |
|  | xylanase inhibitor | | | LOC_Os05g33410 | | 0.03017 | 2.20938 | |  | |  |
|  | OsPOP21 - Putative Prolyl Oligopeptidase homologue | | | LOC_Os10g28020 | | 0.01911 | 2.14218 | |  | |  |
|  | OsPOP7 - Putative Prolyl Oligopeptidase homologue | | | LOC_Os03g19410 | | 0.01508 | 2.03723 | |  | |  |
|  | DnaK family protein | | | LOC_Os02g53420 | | 0.00537 | 2.02210 | |  | |  |
|  | mitochondrial-processing peptidase subunit alpha | | | LOC_Os01g09560 | | 0.00402 | 2.01439 | |  | |  |
|  | LTPL113 - Protease inhibitor/seed storage/LTP family protein precursor | | | LOC_Os02g44320 | | 0.04716 | 1.99075 | |  | |  |
|  | aminopeptidase | | | LOC_Os08g44860 | | 0.00004 | 1.93223 | |  | |  |
|  | T-complex protein | | | LOC_Os10g32550 | | 0.01499 | 1.92856 | |  | |  |
|  | OsSub11 - Putative Subtilisin homologue | | | LOC_Os01g64860 | | 0.00364 | 1.84841 | |  | |  |
|  | T-complex protein | | | LOC_Os03g64210 | | 0.00425 | 1.81205 | |  | |  |
|  | T-complex protein | | | LOC_Os02g01280 | | 0.03222 | 1.78053 | |  | |  |
|  | oryzain gamma chain precursor | | | LOC_Os09g27030 | | 0.02465 | 1.70961 | |  | |  |
|  | ATP-dependent Clp protease ATP-binding subunit clpA | | | LOC_Os04g32560 | | 0.00027 | 1.65960 | |  | |  |
|  | OsClp3 - Putative Clp protease homologue | | | LOC_Os02g42290 | | 0.00001 | 1.63585 | |  | |  |
|  | SET domain containing protein | | | LOC_Os03g19480 | | 0.01076 | 1.61312 | |  | |  |
|  | peptidyl-prolyl cis-trans isomerase, FKBP-type | | | LOC_Os06g45340 | | 0.00887 | 1.59967 | |  | |  |
|  | oryzain alpha chain precursor | | | LOC_Os04g55650 | | 0.00093 | 1.55626 | |  | |  |
|  | peptidyl-prolyl cis-trans isomerase, FKBP-type | | | LOC_Os08g42850 | | 0.00005 | 1.55382 | |  | |  |
|  | DnaK family protein | | | LOC_Os05g35400 | | 0.01756 | 1.55253 | |  | |  |
| General metabolic processes | glycine-rich protein 2 | | | LOC_Os02g02870 | | 0.02765 | 4.67665 | |  | |  |
|  | copine | | | LOC_Os08g38600 | | 0.02120 | 3.51907 | |  | |  |
|  | CBS domain containing membrane protein | | | LOC_Os03g52690 | | 0.02765 | 2.94738 | |  | |  |
|  | transferase family protein | | | LOC_Os08g01980 | | 0.00037 | 2.80737 | |  | |  |
|  | 14-3-3 protein | | | LOC_Os08g37490 | | 0.00341 | 2.67270 | |  | |  |
|  | dehydrogenase E1 | | | LOC_Os02g50620 | | 0.00590 | 2.58103 | |  | |  |
|  | SOR/SNZ family protein | | | LOC_Os10g01080 | | 0.01603 | 2.27388 | |  | |  |
|  | dienelactone hydrolase family protein | | | LOC_Os01g34700 | | 0.02105 | 2.20436 | |  | |  |
|  | HAD-superfamily hydrolase, subfamily IA, variant 3 containing protein | | | LOC_Os03g19760 | | 0.02171 | 1.97891 | |  | |  |
|  | methyltransferase domain containing protein | | | LOC_Os12g42090 | | 0.01993 | 1.84373 | |  | |  |
|  | amine oxidase precursor | | | LOC_Os04g20164 | | 0.00577 | 1.83648 | |  | |  |
|  | dehydrogenase | | | LOC_Os06g46372 | | 0.04706 | 1.83161 | |  | |  |
|  | 2Fe-2S iron-sulfur cluster binding domain containing protein | | | LOC_Os07g30670 | | 0.00300 | 1.74025 | |  | |  |
|  | inositol-1-monophosphatase | | | LOC_Os02g07350 | | 0.00066 | 1.73214 | |  | |  |
|  | pyruvate phosphate dikinase | | | LOC_Os05g33570 | | 0.00160 | 1.72045 | |  | |  |
|  | NADPH-dependent FMN reductase domain containing protein | | | LOC_Os01g57570 | | 0.00361 | 1.71242 | |  | |  |
|  | SOR/SNZ family protein | | | LOC_Os07g01020 | | 0.01016 | 1.66036 | |  | |  |
|  | transferase family protein | | | LOC_Os01g24790 | | 0.00594 | 1.65756 | |  | |  |
|  | dehydrogenase | | | LOC_Os08g43190 | | 0.02360 | 1.57246 | |  | |  |
|  | receptor-like protein kinase homolog RK20-1 | | | LOC_Os12g41410 | | 0.00000 | - | | x | |  |
| Carbohydrate metabolism and energy production | **hexokinase** | | | **LOC_Os07g09890** | | **0.00052** | **19.47512** | |  | |  |
|  | ATP synthase delta chain | | | LOC_Os07g31300 | | 0.01715 | 6.42940 | |  | |  |
|  | glucose-1-phosphate adenylyltransferase large subunit | | | LOC_Os08g25734 | | 0.00204 | 4.37946 | |  | |  |
|  | ATP synthase like protein | | | LOC_Os05g35320 | | 0.00084 | 3.50433 | |  | |  |
|  | pyruvate kinase | | | LOC_Os04g58110 | | 0.02126 | 3.43661 | |  | |  |
|  | galactose mutarotase-like | | | LOC_Os03g06230 | | 0.00067 | 3.19193 | |  | |  |
|  | triosephosphate isomerase | | | LOC_Os01g62420 | | 0.00141 | 2.96071 | |  | |  |
|  | aconitate hydratase protein | | | LOC_Os03g04410 | | 0.00449 | 2.32994 | |  | |  |
|  | cytochrome c | | | LOC_Os05g34770 | | 0.02706 | 1.99490 | |  | |  |
|  | glycosyl hydrolase | | | LOC_Os01g19750 | | 0.01018 | 1.81273 | |  | |  |
|  | lactate/malate dehydrogenase | | | LOC_Os05g49880 | | 0.01119 | 1.78932 | |  | |  |
|  | glucose-1-phosphate adenylyltransferase large subunit | | | LOC_Os08g25734 | | 0.00045 | 1.69538 | |  | |  |
|  | ATP synthase | | | LOC_Os01g51380 | | 0.00220 | 1.68580 | |  | |  |
|  | lactate/malate dehydrogenase | | | LOC_Os01g46070 | | 0.01706 | 1.65367 | |  | |  |
|  | enolase | | | LOC_Os06g04510 | | 0.00535 | 1.64711 | |  | |  |
|  | fructose-1,6-bisphosphatase | | | LOC_Os03g16050 | | 0.01199 | 1.58790 | |  | |  |
|  | NAD dependent epimerase/dehydratase family protein | | | LOC_Os07g11110 | | 0.00133 | 1.57938 | |  | |  |
|  | AAA-type ATPase | | | LOC_Os11g47970 | | 0.00187 | 1.51262 | |  | |  |
| Oxidative stress-related | peroxidase precursor | | | LOC_Os03g55410 | | 0.00290 | 12.33350 | |  | |  |
|  | peroxidase precursor | | | LOC_Os03g22010 | | 0.00734 | 4.50023 | |  | |  |
|  | thioredoxin domain-containing protein 17 | | | LOC_Os06g21550 | | 0.02561 | 3.64432 | |  | |  |
|  | oxidoreductase, aldo/keto reductase family protein | | | LOC_Os07g04990 | | 0.00072 | 2.49270 | |  | |  |
|  | oxidoreductase, aldo/keto reductase family protein | | | LOC_Os05g38230 | | 0.00474 | 2.40761 | |  | |  |
|  | peroxidase precursor | | | LOC_Os05g04380 | | 0.01584 | 2.19971 | |  | |  |
|  | peroxidase precursor | | | LOC_Os09g29490 | | 0.00854 | 1.95827 | |  | |  |
|  | glutathione S-transferase | | | LOC_Os03g04250 | | 0.01521 | 1.95598 | |  | |  |
|  | copper/zinc superoxide dismutase | | | LOC_Os08g44770 | | 0.00236 | 1.78023 | |  | |  |
|  | peroxiredoxin | | | LOC_Os02g09940 | | 0.00139 | 1.76272 | |  | |  |
|  | peroxidase precursor | | | LOC_Os01g22230 | | 0.02832 | 1.68168 | |  | |  |
|  | hydroxyacid oxidase 1 | | | LOC_Os04g53210 | | 0.00019 | 1.61732 | |  | |  |
|  | glutathione S-transferase | | | LOC_Os03g04260 | | 0.00591 | 1.61497 | |  | |  |
|  | glutathione S-transferase | | | LOC_Os03g04240 | | 0.00529 | 1.55622 | |  | |  |
|  | peroxiredoxin | | | LOC_Os06g09610 | | 0.00038 | 1.50621 | |  | |  |
|  | rieske domain containing protein | | | LOC_Os11g13850 | | 0.02746 | 1.50071 | |  | |  |
| Stress response | stress responsive protein | | | LOC_Os01g01450 | | 0.00646 | 8.90599 | |  | |  |
|  | NB-ARC domain containing protein | | | LOC_Os11g45190 | | 0.01310 | 4.32706 | |  | |  |
|  | hsp20/alpha crystallin family protein | | | LOC_Os10g07210 | | 0.00101 | 3.64822 | |  | |  |
|  | osmotin | | | LOC_Os12g38170 | | 0.00570 | 2.59802 | |  | |  |
|  | salt stress root protein RS1 | | | LOC_Os01g13210 | | 0.00033 | 2.16218 | |  | |  |
|  | heat shock protein | | | LOC_Os09g29840 | | 0.00421 | 2.03664 | |  | |  |
|  | thaumatin family domain containing protein | | | LOC_Os12g38120 | | 0.01042 | 1.96427 | |  | |  |
|  | remorin | | | LOC_Os04g45070 | | 0.00078 | 1.66891 | |  | |  |
| Photosynthesis | thylakoid lumenal protein | | | LOC_Os02g42960 | | 0.00083 | 2.05717 | |  | |  |
|  | chlorophyll A-B binding protein | | | LOC_Os09g17740 | | 0.02271 | 1.78440 | |  | |  |
|  | phosphoenolpyruvate carboxylase | | | LOC_Os09g14670 | | 0.00721 | 1.77008 | |  | |  |
|  | thylakoid lumenal 20 kDa protein | | | LOC_Os01g59090 | | 0.00273 | 1.71783 | |  | |  |
|  | photosystem II 11 kD protein | | | LOC_Os03g21560 | | 0.01478 | 1.61197 | |  | |  |
|  | chlorophyll A-B binding protein | | | LOC_Os07g37550 | | 0.00572 | 1.52931 | |  | |  |
|  | chlorophyll A-B binding protein | | | LOC_Os02g10390 | | 0.00101 | 1.50500 | |  | |  |
|  | chlorophyll A-B binding protein | | | LOC_Os01g64960 | | 0.02451 | 1.50409 | |  | |  |
| Translation-related | ribosomal protein S2 | | | LOC_Os12g34092 | | 0.00006 | 1.90335 | |  | |  |
|  | 50S ribosomal protein L31 | | | LOC_Os01g44210 | | 0.02173 | 1.80961 | |  | |  |
|  | ribosomal protein S6 | | | LOC_Os03g62630 | | 0.01237 | 1.70969 | |  | |  |
|  | L1P family of ribosomal proteins domain containing protein | | | LOC_Os05g32220 | | 0.00438 | 1.56541 | |  | |  |
|  | 60S ribosomal protein L19-3 | | | LOC_Os03g21940 | | 0.01016 | 1.51035 | |  | |  |
| Transcription-related | transcription elongation factor | | | LOC_Os11g06650 | | 0.00474 | 2.73208 | |  | |  |
|  | HMG-Y-related protein A | | | LOC_Os09g23730 | | 0.00801 | 2.62167 | |  | |  |
|  | KH domain containing protein | | | LOC_Os03g42900 | | 0.02313 | 1.96087 | |  | |  |
|  | KH domain-containing protein | | | LOC_Os02g13130 | | 0.00177 | 1.85828 | |  | |  |
| Amino acid metabolism | 2-isopropylmalate synthase B | | | LOC_Os12g04440 | | 0.00198 | 2.39560 | |  | |  |
|  | glycine dehydrogenase | | | LOC_Os06g40940 | | 0.00216 | 1.72954 | |  | |  |
|  | glycine dehydrogenase | | | LOC_Os01g51410 | | 0.00726 | 1.69429 | |  | |  |
|  | cysteine synthase | | | LOC_Os01g74650 | | 0.00760 | 1.51152 | |  | |  |
| DNA structure maintenance | Histone H3 | | | LOC_Os04g34240 | | 0.04405 | 2.08855 | |  | |  |
|  | Core histone H2A/H2B/H3/H4 domain containing protein | | | LOC_Os03g17084 | | 0.02387 | 1.74328 | |  | |  |
|  | Core histone H2A/H2B/H3/H4 domain containing protein | | | LOC_Os09g26340 | | 0.02098 | 1.72931 | |  | |  |
| Transport-related | aquaporin protein | | | LOC_Os02g57720 | | 0.00588 | 3.59476 | |  | |  |
| Lipid metabolism | enoyl-acyl-carrier-protein reductase NADH | | | LOC_Os08g23810 | | 0.00997 | 2.72317 | |  | |  |
| Protein structure maintenance | PAP fibrillin family domain containing protein | | | LOC_Os09g04790 | | 0.00037 | 1.91762 | |  | |  |
| Hormone-related | auxin-binding protein 4 precursor | | | LOC_Os12g34460 | | 0.04342 | 1.68560 | |  | |  |
| Others | C2 domain containing protein | | | LOC_Os02g58230 | | 0.00275 | 7.83302 | |  | |  |
|  | flavonol-3-O-glycoside-7-O-glucosyltransferase 1 | | | LOC_Os01g08090 | | 0.00065 | 4.23491 | |  | |  |
|  | membrane-associated 30 kDa protein | | | LOC_Os01g67000 | | 0.00020 | 2.46600 | |  | |  |
|  | OsMADS15 - MADS-box family gene with MIKCc type-box | | | LOC_Os07g01820 | | 0.04438 | 4.16631 | |  | |  |
|  | Cupin domain containing protein | | | LOC_Os01g14670 | | 0.00324 | 1.75700 | |  | |  |
|  | Cupin domain containing protein | | | LOC_Os08g35760 | | 0.00518 | 1.54280 | |  | |  |
| Unknown | hypothetical protein | | | LOC_Os04g22890 | | 0.00004 | 3.85200 | |  | |  |
|  | expressed protein | | | LOC_Os02g28680 | | 0.00196 | 3.35994 | |  | |  |
|  | protein of unknown function domain containing protein | | | LOC_Os01g07810 | | 0.02661 | 2.32604 | |  | |  |
|  | hypothetical protein | | | LOC_Os12g13530 | | 0.00644 | 2.09710 | |  | |  |
|  | expressed protein | | | LOC_Os01g67080 | | 0.00726 | 1.88450 | |  | |  |
|  | expressed protein | | | LOC_Os03g64020 | | 0.04427 | 1.86787 | |  | |  |
|  | expressed protein | | | LOC_Os03g60740 | | 0.02464 | 1.78890 | |  | |  |
|  | expressed protein | | | LOC_Os02g48480 | | 0.01817 | 1.74601 | |  | |  |
|  | expressed protein | | | LOC_Os08g16570 | | 0.00016 | 1.71326 | |  | |  |
|  | expressed protein | | | LOC_Os03g61090 | | 0.04468 | 1.59465 | |  | |  |
|  | expressed protein | | | LOC_Os03g53419 | | 0.00004 | 1.54675 | |  | |  |
|  | LYK | | | LOC_Os11g35330 | | 0.03450 | 1.54235 | |  | |  |
|  | expressed protein | | | LOC_Os09g17660 | | 0.00915 | 1.50414 | |  | |  |

**Obs: Bold and underlined sequences were confirmed by RT-qPCR.**
