# Supplementary material for: Unraveling Rice Tolerance Mechanisms Against Schizotetranychus oryzae Mite Infestation
Source: Front Plant Sci. 2018 Sep 18;9:1341. doi: 10.3389/fpls.2018.01341 (PMC6153315; doi:10.3389/fpls.2018.01341)
Supplement: TABLE S4 — Differentially abundant proteins in control condition (susceptible Puitá INTA-CL × tolerant IRGA 423). [file Table_4.DOCX]

| **Control Puitá INTA-CL x IRGA 423 - Proteins unique or more expressed in Puitá INTA-CL leaves** | | | | | | |
| --- | --- | --- | --- | --- | --- | --- |
| **Functional categories** | **Description** | **Locus** | | **ANOVA** | **Fold change IRGA 423/Puitá INTA-CL** | **Unique to Puitá INTA-CL** |
| Translation-related | chloroplast 50S ribosomal protein L16 | LOC_Os04g16826 | | 0.01142 | 0.66006 |  |
|  | elongation factor protein | LOC_Os07g46750 | | 0.01011 | 0.64224 |  |
|  | 40S ribosomal protein S3-1 | LOC_Os03g38000 | | 0.00914 | 0.63476 |  |
|  | ribosomal protein S2 | LOC_Os03g08440 | | 0.00555 | 0.62610 |  |
|  | 40S ribosomal protein S27 | LOC_Os04g27860 | | 0.03814 | 0.62565 |  |
|  | 60S acidic ribosomal protein | LOC_Os06g48780 | | 0.01630 | 0.60699 |  |
|  | ribosomal protein S6 | LOC_Os03g62630 | | 0.00786 | 0.57758 |  |
|  | nascent polypeptide-associated complex subunit alpha | LOC_Os03g02960 | | 0.01538 | 0.57465 |  |
|  | elongation factor 1-gamma | LOC_Os02g12800 | | 0.00806 | 0.57254 |  |
|  | 60S acidic ribosomal protein | LOC_Os08g02340 | | 0.00321 | 0.55141 |  |
|  | 60S ribosomal protein L19-3 | LOC_Os03g21940 | | 0.00096 | 0.41664 |  |
|  | 50S ribosomal protein L31 | LOC_Os01g44210 | | 0.00623 | 0.39528 |  |
|  | tRNA synthetases class II domain containing protein | LOC_Os01g27520 | | 0.00054 | 0.36146 |  |
|  | ribosomal protein L4 | LOC_Os03g15870 | | 0.00428 | 0.36073 |  |
|  | L1P family of ribosomal proteins domain containing protein | LOC_Os05g32220 | | 0.00030 | 0.35676 |  |
|  | ribosomal protein L4 | LOC_Os03g58204 | | 0.01891 | 0.29925 |  |
|  | elongation factor | LOC_Os01g53900 | | 0.00107 | 0.19909 |  |
| Protein modification/degradation | ubiquitin family protein | LOC_Os08g08760 | | 0.00155 | 0.63721 |  |
|  | T-complex protein | LOC_Os10g32550 | | 0.01416 | 0.63202 |  |
|  | ATP-dependent Clp protease ATP-binding subunit clpA | LOC_Os04g32560 | | 0.00122 | 0.62282 |  |
|  | peptidyl-prolyl cis-trans isomerase | LOC_Os02g02890 | | 0.00212 | 0.62098 |  |
|  | DnaK family protein | LOC_Os02g53420 | | 0.01951 | 0.61624 |  |
|  | DnaK family protein | LOC_Os05g38530 | | 0.02891 | 0.61231 |  |
|  | OsSub11 - Putative Subtilisin homologue | LOC_Os01g64860 | | 0.00206 | 0.59992 |  |
|  | aminopeptidase | LOC_Os08g44860 | | 0.00044 | 0.59727 |  |
|  | uncharacterized kinase mug58 | LOC_Os01g48990 | | 0.00344 | 0.59026 |  |
|  | OsPOP7 - Putative Prolyl Oligopeptidase homologue | LOC_Os03g19410 | | 0.02141 | 0.58992 |  |
|  | peptidyl-prolyl cis-trans isomerase CYP37 | LOC_Os07g37830 | | 0.00421 | 0.54842 |  |
|  | oryzain gamma chain precursor | LOC_Os09g27030 | | 0.01476 | 0.53227 |  |
|  | peptidyl-prolyl cis-trans isomerase, FKBP-type | LOC_Os02g51570 | | 0.01989 | 0.50109 |  |
|  | serine/threonine protein phosphatase 2A 55 kDa regulatory subunit B | LOC_Os02g40454 | | 0.02046 | 0.48656 |  |
|  | mitochondrial-processing peptidase subunit | LOC_Os03g11410 | | 0.00653 | 0.38395 |  |
|  | 4-nitrophenylphosphatase | LOC_Os09g08660 | | 0.00001 | 0.15864 |  |
|  | cysteine proteinase EP-B 1 precursor | LOC_Os09g39100 | | 0.01220 | 0.07988 |  |
| General metabolic processes | ras-related protein | LOC_Os09g35860 | | 0.03152 | 0.65817 |  |
|  | methylisocitrate lyase 2 | LOC_Os04g31700 | | 0.00488 | 0.64797 |  |
|  | dihydrolipoyl dehydrogenase | LOC_Os05g06750 | | 0.02520 | 0.62890 |  |
|  | dihydrolipoyllysine-residue succinyltransferase component of 2-oxoglutarate dehydrogenase complex | LOC_Os04g32330 | | 0.01630 | 0.60118 |  |
|  | dehydrogenase E1 | LOC_Os02g50620 | | 0.00116 | 0.59904 |  |
|  | HAD-superfamily hydrolase, subfamily IA, variant 3 containing protein | LOC_Os03g19760 | | 0.00243 | 0.59723 |  |
|  | dehydrogenase | LOC_Os09g23550 | | 0.00788 | 0.54974 |  |
|  | HAD superfamily phosphatase | LOC_Os01g09540 | | 0.00017 | 0.53917 |  |
|  | aminotransferase, classes I and II, domain containing protein | LOC_Os03g18810 | | 0.04004 | 0.47105 |  |
|  | copine | LOC_Os08g38600 | | 0.04859 | 0.39751 |  |
|  | CBS domain containing membrane protein | LOC_Os02g57280 | | 0.01908 | 0.35132 |  |
|  | soluble inorganic pyrophosphatase | LOC_Os02g47600 | | 0.00259 | 0.33652 |  |
|  | glycine-rich protein 2 | LOC_Os02g02870 | | 0.00894 | 0.20529 |  |
|  | receptor-like protein kinase homolog RK20-1 | LOC_Os12g41410 | | 0.00014 | - | x |
| Carbohydrate metabolism and energy production | glyceraldehyde-3-phosphate dehydrogenase | LOC_Os04g40950 | | 0.04545 | 0.66355 |  |
|  | aconitate hydratase protein | LOC_Os03g04410 | | 0.00063 | 0.63088 |  |
|  | galactose mutarotase-like | LOC_Os03g06230 | | 0.00505 | 0.61706 |  |
|  | ATP synthase like protein | LOC_Os05g35320 | | 0.01128 | 0.60726 |  |
|  | lactate/malate dehydrogenase | LOC_Os01g46070 | | 0.01580 | 0.59441 |  |
|  | aconitate hydratase protein | LOC_Os08g09200 | | 0.00018 | 0.55795 |  |
|  | triosephosphate isomerase | LOC_Os01g62420 | | 0.00116 | 0.54853 |  |
|  | glucose-1-phosphate adenylyltransferase large subunit | LOC_Os08g25734.1 | | 0.00037 | 0.54616 |  |
|  | fructose-bisphospate aldolase isozyme | LOC_Os10g08022 | | 0.00237 | 0.50872 |  |
|  | glucose-1-phosphate adenylyltransferase large subunit | LOC_Os08g25734.2 | | 0.00009 | 0.37351 |  |
|  | **hexokinase** | **LOC_Os07g09890** | | **0.00023** | **0.23978** |  |
| Oxidative stress-related | glutathione S-transferase | LOC_Os03g04250 | | 0.03589 | 0.65699 |  |
|  | peroxidase precursor | LOC_Os01g73170 | | 0.00131 | 0.62574 |  |
|  | peroxiredoxin | LOC_Os02g09940 | | 0.00101 | 0.62355 |  |
|  | copper/zinc superoxide dismutase | LOC_Os03g22810 | | 0.00599 | 0.62317 |  |
|  | glutathione S-transferase | LOC_Os09g29200 | | 0.00209 | 0.37429 |  |
|  | peroxidase precursor | LOC_Os03g55410 | | 0.00704 | 0.22451 |  |
|  | thioredoxin domain-containing protein 17 | LOC_Os06g21550 | | 0.00977 | 0.17381 |  |
| Stress response | remorin | LOC_Os04g45070 | | 0.00043 | 0.62403 |  |
|  | osmotin | LOC_Os12g38170 | | 0.02125 | 0.57243 |  |
|  | hsp20/alpha crystallin family protein | LOC_Os10g07210 | | 0.00504 | 0.43365 |  |
|  | heat shock protein | LOC_Os08g39140 | | 0.00328 | 0.38479 |  |
|  | stress responsive protein | LOC_Os01g01450 | | 0.03055 | 0.28438 |  |
| Amino acid metabolism | glycine dehydrogenase | LOC_Os01g51410 | | 0.01717 | 0.65439 |  |
|  | 2-isopropylmalate synthase B | LOC_Os12g04440 | | 0.00661 | 0.63147 |  |
|  | ornithine carbamoyltransferase | LOC_Os02g47590 | | 0.00319 | 0.62958 |  |
|  | argininosuccinate synthase | LOC_Os12g13320 | | 0.02534 | 0.52704 |  |
| Photosynthesis | chlorophyll A-B binding protein | LOC_Os09g17740 | | 0.01104 | 0.65309 |  |
|  | thylakoid lumenal protein | LOC_Os02g42960 | | 0.00344 | 0.61186 |  |
|  | thylakoid lumenal protein | LOC_Os01g05080 | | 0.00559 | 0.54633 |  |
|  | calvin cycle protein CP12 | LOC_Os03g19380 | | 0.00883 | 0.51349 |  |
| Transcription-related | KH domain-containing protein | LOC_Os02g13130 | | 0.00011 | 0.63417 |  |
|  | HMG-Y-related protein A | LOC_Os09g23730 | | 0.01221 | 0.55924 |  |
| Transport-related | aquaporin protein | LOC_Os02g57720 | | 0.00829 | 0.58180 |  |
|  | outer mitochondrial membrane porin | LOC_Os05g45950 | | 0.00162 | 0.56150 |  |
| Protein structure maintenance | PAP fibrillin family domain containing protein | LOC_Os09g04790 | | 0.00180 | 0.64039 |  |
| DNA structure maintenance | SET domain containing protein | LOC_Os03g19480 | | 0.00983 | 0.63421 |  |
| Cell structure and cell division | profilin domain containing protein | LOC_Os06g05880 | | 0.00718 | 0.54762 |  |
| Lipid metabolism | 3-ketoacyl-CoA thiolase | LOC_Os02g57260 | | 0.00145 | 0.48992 |  |
| Secondary metabolism | chalcone synthase | LOC_Os01g41834 | | 0.03281 | 0.36969 |  |
| Others | actin | LOC_Os03g61970 | | 0.04287 | 0.60292 |  |
|  | Cupin domain containing protein | LOC_Os03g48770 | | 0.02164 | 0.53692 |  |
|  | C2 domain containing protein | LOC_Os02g58230 | | 0.00058 | 0.33677 |  |
| Unknown | expressed protein | LOC_Os10g22110 | | 0.01035 | 0.66155 |  |
|  | LYK | LOC_Os11g35330 | | 0.03083 | 0.62500 |  |
|  | expressed protein | LOC_Os03g60740 | | 0.00680 | 0.56879 |  |
|  | expressed protein | LOC_Os03g53419 | | 0.00125 | 0.51901 |  |
|  | hypothetical protein | LOC_Os04g22890 | | 0.01604 | 0.46479 |  |
|  | protein of unknown function domain containing protein | LOC_Os01g07810 | | 0.00331 | 0.20353 |  |
| **Control Puitá INTA-CL x IRGA 423 - Proteins unique or more expressed in IRGA 423 leaves** | | | | | | |
| **Functional categories** | **Description** | | **Locus** | **ANOVA** | **Fold change IRGA 423/Puitá INTA-CL** | |
| Carbohydrate metabolism and energy production | ATP synthase subunit beta | | LOC_Os06g39740 | 0.00010 | 9.38103 | |
|  | **2,3-bisphosphoglycerate-independent phosphoglycerate mutase** | | **LOC_Os05g40420** | 0.00000 | 3.23467 | |
|  |  | |  |  |  | |
|  | starch synthase | | LOC_Os07g22930 | 0.00814 | 1.81241 | |
|  | ATP synthase | | LOC_Os01g49190 | 0.00898 | 1.53064 | |
|  | NAD binding domain of 6-phosphogluconate dehydrogenase containing protein | | LOC_Os01g39270 | 0.01207 | 1.52578 | |
| General metabolic processes | receptor-like protein kinase 2 precursor | | LOC_Os02g06280 | 0.01037 | 12.07468 | |
|  | STRUBBELIG-RECEPTOR FAMILY 6 precursor | | LOC_Os03g51040 | 0.00550 | 8.06700 | |
|  | kelch repeat protein | | LOC_Os04g40740 | 0.00120 | 5.51093 | |
|  | bifunctional 3-phosphoadenosine 5-phosphosulfate synthetase | | LOC_Os04g02050 | 0.02665 | 1.72588 | |
|  | dehydrogenase | | LOC_Os09g23540 | 0.01592 | 1.66529 | |
| Oxidative stress-related | rubredoxin family protein | | LOC_Os08g23410 | 0.00005 | 7.57667 | |
|  | peroxidase precursor | | LOC_Os01g22249 | 0.04871 | 2.86640 | |
|  | OsGrx_S16 - glutaredoxin subgroup II | | LOC_Os12g07650 | 0.00170 | 2.32038 | |
| Stress response | NBS-LRR type disease resistance protein Rps1-k-2 | | LOC_Os12g10180 | 0.00187 | 3.91302 | |
|  | universal stress protein domain containing protein | | LOC_Os03g53900 | 0.00112 | 2.13675 | |
|  | phosducin-like protein 3 | | LOC_Os01g08960 | 0.01643 | 1.63378 | |
| Translation-related | ribosomal protein | | LOC_Os02g06700 | 0.00051 | 1.94188 | |
|  | G-patch domain containing protein | | LOC_Os03g14860 | 0.02906 | 1.58294 | |
|  | elongation factor | | LOC_Os02g32030 | 0.00055 | 1.56090 | |
| Cell structure and cell division | myosin-Vb | | LOC_Os07g37560 | 0.04529 | 3.21819 | |
|  | kinesin motor domain containing protein | | LOC_Os03g53920 | 0.02206 | 2.50514 | |
| Photosynthesis | photosystem II P680 chlorophyll A apoprotein | | LOC_Os09g04680 | 0.01699 | 4.63638 | |
|  | PsbP | | LOC_Os03g17174 | 0.00160 | 2.75817 | |
| Protein modification/degradation | cell division protease ftsH homolog 4 | | LOC_Os04g39190 | 0.00020 | 2.21543 | |
|  | AAA-type ATPase family protein | | LOC_Os04g56320 | 0.00290 | 1.81321 | |
| Transcription-related | KH domain containing protein | | LOC_Os03g60110 | 0.00029 | 36.85924 | |
| Lipid metabolism | enoyl-CoA hydratase/isomerase family protein | | LOC_Os06g39344 | 0.00018 | 10.69030 | |
| Transport-related | ABC transporter, ATP-binding protein | | LOC_Os03g21490 | 0.00032 | 2.35988 | |
| Storage-related | glutelin | | LOC_Os02g25640 | 0.01080 | 2.16740 | |
| Secondary metabolism | terpene synthase | | LOC_Os04g26980 | 0.03200 | 1.81273 | |
| DNA structure maintenance | MAR-binding filament-like protein 1 | | LOC_Os05g08790 | 0.02703 | 1.57659 | |
| Others | ATP11 protein | | LOC_Os02g20860 | 0.00041 | 2.80416 | |
|  | metallo-beta-lactamase family protein | | LOC_Os03g21460 | 0.00633 | 2.23477 | |
|  | sex determination protein tasselseed-2 | | LOC_Os04g33240 | 0.01173 | 2.11773 | |
|  | PPR repeat domain containing protein | | LOC_Os07g31310 | 0.00778 | 1.86930 | |
|  | actin | | LOC_Os01g64630 | 0.00542 | 1.58633 | |
| Unknown | expressed protein | | LOC_Os04g57020 | 0.00892 | 2.91455 | |
|  | expressed protein | | LOC_Os05g49080 | 0.01577 | 2.32431 | |
|  | expressed protein | | LOC_Os03g61090 | 0.02441 | 2.03735 | |
|  | expressed protein | | LOC_Os04g01540 | 0.00042 | 1.92321 | |

**Obs: Bold and underlined sequences were confirmed by RT-qPCR.**
