# Supplementary material for: Unraveling Rice Tolerance Mechanisms Against Schizotetranychus oryzae Mite Infestation
Source: Front Plant Sci. 2018 Sep 18;9:1341. doi: 10.3389/fpls.2018.01341 (PMC6153315; doi:10.3389/fpls.2018.01341)
Supplement: TABLE S5 — Differentially abundant proteins in infested condition (susceptible Puitá INTA-CL × tolerant IRGA 423). [file Table_5.DOCX]

| **Infested Puitá INTA-CL x IRGA 423 - Proteins more expressed in Puitá INTA-CL leaves** | | | | | |
| --- | --- | --- | --- | --- | --- |
| **Functional categories** | **Description** | | **Locus** | **ANOVA** | **Fold change IRGA 423/Puitá INTA-CL** |
| Translation-related | elongation factor 1-gamma | | LOC_Os02g12800 | 0.00037 | 0.61642 |
|  | G-patch domain containing protein | | LOC_Os03g14860 | 0.00383 | 0.58706 |
|  | elongation factor protein | | LOC_Os07g42300 | 0.03013 | 0.57748 |
|  | elongation factor | | LOC_Os02g32030 | 0.03207 | 0.52431 |
|  | ribosomal protein | | LOC_Os02g06700 | 0.04733 | 0.24391 |
| Transport-related | OsTIL-1 Temperature-induced lipocalin-1 | | LOC_Os02g39930 | 0.00406 | 0.66631 |
|  | OsCHL Chloroplastic lipocalin | | LOC_Os04g53490 | 0.00443 | 0.64808 |
|  | atATG18b | | LOC_Os02g54910 | 0.00185 | 0.46143 |
|  | cation efflux family protein | | LOC_Os04g23180 | 0.01963 | 0.36340 |
| Protein modification/degradation | OsFtsH6 FtsH protease, homologue of AtFtsH6 | | LOC_Os06g12370 | 0.03682 | 0.44076 |
|  | peptidyl-prolyl cis-trans isomerase, FKBP-type | | LOC_Os02g52290 | 0.00959 | 0.32855 |
|  | DnaK family protein | | LOC_Os03g60620 | 0.03277 | 0.26071 |
| Carbohydrate metabolism and energy production | NAD binding domain of 6-phosphogluconate dehydrogenase containing protein | | LOC_Os01g39270 | 0.00063 | 0.31854 |
|  | **2,3-bisphosphoglycerate-independent phosphoglycerate mutase** | | **LOC_Os05g40420** | **0.00027** | **0.18132** |
| General metabolic processes | pyruvate, phosphate dikinase | | LOC_Os03g31750 | 0.00698 | 0.65331 |
|  | dehydrogenase | | LOC_Os09g23540 | 0.00670 | 0.57593 |
| Photosynthesis | thylakoid lumenal protein | | LOC_Os01g05080 | 0.04658 | 0.63771 |
|  | PsbP | | LOC_Os08g25900 | 0.04218 | 0.34739 |
| Amino acid metabolism | amine oxidase, flavin-containing, domain containing protein | | LOC_Os09g20284 | 0.03891 | 0.65573 |
| Cell structure and cell division | profilin domain containing protein | | LOC_Os06g05880 | 0.02039 | 0.62474 |
| Hormone-related | gibberellin receptor | | LOC_Os09g28630 | 0.04734 | 0.60181 |
| Oxidative stress-related | OsGrx_S16 - glutaredoxin subgroup II | | LOC_Os12g07650 | 0.02075 | 0.53051 |
| Stress response | phosducin-like protein 3 | | LOC_Os01g08960 | 0.00185 | 0.44903 |
| Storage-related | glutelin | | LOC_Os02g25640 | 0.02528 | 0.25692 |
| Others | metallo-beta-lactamase family protein | | LOC_Os03g21460 | 0.01355 | 0.22550 |
|  | endoribonuclease | | LOC_Os07g33240 | 0.03050 | 0.37883 |
| Unknown | expressed protein | | LOC_Os07g10620 | 0.03269 | 0.39709 |
|  | expressed protein | | LOC_Os05g49080 | 0.00410 | 0.18992 |
| **Infested Puitá INTA-CL x IRGA 423 - Proteins more expressed in IRGA 423 leaves** | | | | | |
| **Functional categories** | **Description** | **Locus** | | **ANOVA** | **Fold change IRGA 423/Puitá INTA-CL** |
| Carbohydrate metabolism and energy production | **hexokinase** | **LOC_Os07g09890** | | 0.02196 | 2.86774 |
|  | triosephosphate isomerase | LOC_Os01g62420 | | 0.01034 | 1.98390 |
|  | galactose mutarotase-like | LOC_Os03g06230 | | 0.01231 | 1.76726 |
|  | cytochrome c | LOC_Os05g34770 | | 0.04635 | 1.65711 |
|  | ATP synthase like protein | LOC_Os05g35320 | | 0.02640 | 1.65353 |
|  | aconitate hydratase protein | LOC_Os03g04410 | | 0.04882 | 1.52538 |
| General metabolic processes | CBS domain containing membrane protein | LOC_Os03g52690 | | 0.00128 | 1.99770 |
|  | transferase family protein | LOC_Os08g01980 | | 0.00078 | 1.85612 |
|  | dehydrogenase E1 | LOC_Os02g50620 | | 0.03922 | 1.73494 |
|  | O-methyltransferase | LOC_Os08g06100 | | 0.02426 | 1.66223 |
|  | dihydrolipoyl dehydrogenase 1 | LOC_Os01g22520 | | 0.02032 | 1.59746 |
|  | copine | LOC_Os08g38600 | | 0.00897 | 1.58527 |
| Protein modification/degradation | serine/threonine protein phosphatase 2A regulatory subunit B | LOC_Os02g40454 | | 0.01507 | 4.10635 |
|  | 4-nitrophenylphosphatase | LOC_Os09g08660 | | 0.01213 | 3.28919 |
|  | T-complex protein | LOC_Os10g32550 | | 0.01998 | 1.63091 |
|  | peptidyl-prolyl cis-trans isomerase, FKBP-type | LOC_Os02g51570 | | 0.04455 | 1.57625 |
| DNA structure maintenance | Core histone H2A/H2B/H3/H4 domain containing protein | LOC_Os03g17084 | | 0.02670 | 1.72524 |
|  | Core histone H2A/H2B/H3/H4 domain containing protein | LOC_Os09g26340 | | 0.03247 | 1.60730 |
| Oxidative stress-related | peroxidase precursor | LOC_Os03g22010 | | 0.00307 | 2.56997 |
| Photosynthesis | Calvin cycle protein CP12 | LOC_Os03g19380 | | 0.04025 | 2.09499 |
|  | photosystem I P700 chlorophyll a apoprotein A2 | LOC_Os10g21248 | | 0.01052 | 1.72144 |
| Hormone-related | jasmonate O-methyltransferase | LOC_Os06g21820 | | 0.04290 | 8.59331 |
| Transport-related | aquaporin protein | LOC_Os02g57720 | | 0.01507 | 2.51333 |
| Lipid metabolism | enoyl-acyl-carrier-protein reductase NADH | LOC_Os08g23810 | | 0.01994 | 1.98978 |
| Transcription-related | KH domain containing protein | LOC_Os03g42900 | | 0.03268 | 1.82890 |
| Stress response | hsp20/alpha crystallin family protein | LOC_Os10g07210 | | 0.01790 | 1.69494 |
| Others | flavonol-3-O-glycoside-7-O-glucosyltransferase 1 | LOC_Os01g08090 | | 0.00032 | 3.56282 |
|  | ferredoxin--nitrite reductase | LOC_Os05g42350 | | 0.00053 | 1.67863 |
|  | actin | LOC_Os01g73310 | | 0.02497 | 1.66291 |
| Unknown | expressed protein | LOC_Os01g67080 | | 0.00743 | 1.78867 |
|  | expressed protein | LOC_Os03g61090 | | 0.02960 | 1.52274 |
|  | expressed protein | LOC_Os08g25650 | | 0.01293 | 1.52072 |

**Obs: Bold and underlined sequences were confirmed by RT-qPCR.**
